# Golgi-restored vesicular replenishment retards bone aging and empowers aging bone regeneration

**DOI:** 10.1038/s41413-024-00386-w

**Published:** 2025-02-08

**Authors:** Peisheng Liu, Hao Guo, Xiaoyao Huang, Anqi Liu, Ting Zhu, Chenxi Zheng, Fei Fu, Kaichao Zhang, Shijie Li, Xinyan Luo, Jiongyi Tian, Yan Jin, Kun Xuan, Bingdong Sui

**Affiliations:** 1 State Key Laboratory of Oral & Maxillofacial Reconstruction and Regeneration, National Clinical Research Center for Oral Diseases, Shaanxi International Joint Research Center for Oral Disease, Center for Tissue Engineering, School of Stomatology, The Fourth Military Medical University, Xi’an, 710032 Shaanxi China; 2 State Key Laboratory of Oral & Maxillofacial Reconstruction and Regeneration, National Clinical Research Center for Oral Diseases, Shaanxi Clinical Research Center for Oral Disease, Department of Preventive Dentistry, School of Stomatology, The Fourth Military Medical University, Xi’an, 710032 Shaanxi China; 3 Xi’an Institute of Tissue Engineering and Regenerative Medicine, Xi’an, 710032 Shaanxi China

**Keywords:** Bone, Osteoporosis

## Abstract

Healthy aging is a common goal for humanity and society, and one key to achieving it is the rejuvenation of senescent resident stem cells and empowerment of aging organ regeneration. However, the mechanistic understandings of stem cell senescence and the potential strategies to counteract it remain elusive. Here, we reveal that the aging bone microenvironment impairs the Golgi apparatus thus diminishing mesenchymal stem cell (MSC) function and regeneration. Interestingly, replenishment of cell aggregates-derived extracellular vesicles (CA-EVs) rescues Golgi dysfunction and empowers senescent MSCs through the Golgi regulatory protein Syntaxin 5. Importantly, in vivo administration of CA-EVs significantly enhanced the bone defect repair rate and improved bone mass in aging mice, suggesting their therapeutic value for treating age-related osteoporosis and promoting bone regeneration. Collectively, our findings provide insights into Golgi regulation in stem cell senescence and bone aging, which further highlight CA-EVs as a potential rejuvenative approach for aging bone regeneration.

## Introduction

Age-related osteoporosis is a common skeletal disease in countries with aging populations, which not only jeopardizes human health but also adds to the burden on society.^1^ It is characterized by a reduction in bone mass and a slowed rate of bone regeneration, which leads to an increased susceptibility of damaged bones to fracture.^1,2^ Tissue-resident stem cell senescence is a dominant factor in organismal aging, and bone marrow mesenchymal stem cells (BMSCs), pluripotent stem cells residing in the bone marrow niche, play a key role in maintaining bone homeostasis and regeneration.^3,4^ Studies have shown that the decline in viability and shift in the differentiation direction of BMSCs due to aging is an important cause of the loss of bone regeneration capability, which also contributes to the development of age-related osteoporosis.^5,6^ However, current clinical strategies for age-related osteoporosis and fractures are mainly based on dietary therapies supplemented by exercise or localized physiotherapy, which are conservative, while anti-resorption and anabolic pharmacological therapies represented by bisphosphonates and selective estrogen receptor modulators are taken over a long period of time and have significant side effects.^7^ However, therapeutics targeting the aging of BMSCs have rarely been reported, and the underlying mechanisms and potential molecular targets require further investigation.^8–10^

Stem cell aggregates (CA) is a regenerative technique that promotes normal stem cell function by encouraging high-density stem cells to secrete large amounts of extracellular matrix (ECM), which serves as an excellent cellular scaffold.^11,12^ Furthermore, CA mimics the way stem cells self-organize in the embryonic development, which determines the mode of organogenesis and helps maintain beneficial tissue forming niche.^13^ We have previously shown that CA promote regeneration of multiple tissues/organs in rodents and importantly, in humans.^14–16^ These CA can be established using multiple types of mesenchymal stem cells (MSCs), including BMSCs and stem cells from human exfoliated deciduous teeth (SHED), which have common MSC properties and can be obtained non-invasively with providing a more abundant source for application.^17^ However, the potential regenerative value of CA in the aging condition is not yet investigated. Extracellular vesicles (EVs), emerging messengers for intercellular communication, are capable of modulating the behaviors of recipient cells by transferring bioactive components and are considered to be promising therapeutic agents.^18,19^ Our previous studies have further revealed that CA-derived EVs (CA-EVs) are featured with organogenetic and angiogenic proteins to mediate donor-recipient crosstalk thus effectively promoting tissue/organ regeneration.^14,16,20^ Currently, within the field of aging research, there is a focus on investigating the causes and mechanisms of functional decline of endogenous MSCs, especially in bone aging.^21,22^ Additionally, studies have highlighted the role of EVs in mediating intercellular communication within the aging bone or between bone and extra-bone organs, thereby triggering a cascading response in aging.^23^ Nevertheless, the precise mechanisms by which EVs regulate BMSC senescence in aging remain elusive, and whether CA-EVs retard senescence of tissue-resident stem cells and prevent age-related diseases are not reported.

In this study, we investigated the mechanisms underlying BMSC senescence in bone aging and explored whether CA-EVs can improve bone mass and regeneration with the advanced age. Surprisingly, we discovered that alterations of Golgi apparatus contributed to senescence of resident BMSCs and led to a reduction in the release of endogenous EVs, which has not been previously reported. We further found that locally transplanted CA lost its ability to promote bone regeneration in the aging microenvironment, which was also attributed to impaired structure and function of Golgi. Intriguingly, in-depth analysis suggested that CA-EVs exposed functional surface proteins to assemble the Golgi apparatus, such as Syntaxin 5 (STX5), which helped restore function of senescent BMSCs. Importantly, CA-EV replenishment promoted regeneration of bone defects and counteracted osteoporosis in aging mice. These findings provide the first evidence that Golgi-based vesicular disorders contribute to cell senescence and that CA-EVs effectively mitigate BMSC aging to retard age-related osteoporosis and safeguard aging bone regeneration.

## Results

### Golgi alterations contribute to functional decline of BMSCs in bone aging

To begin, 18-month-old aging mice and their 2-month-old young counterparts were applied in this study. Micro-computed tomography (Micro-CT) and quantitative analysis revealed that the trabecular bone volume over tissue volume (Tb.BV/TV) in aging mice was significantly reduced compared to young mice, and the cortical bone thickness (Ct.Th) was also decreased (Fig. S1A, B). Accordingly, aging BMSCs demonstrated impaired self-renewal capability, as shown by colony-forming unit (CFU) assay with fewer clones and smaller individual clonal areas than young BMSCs (Fig. S1C). Furthermore, Ki67 immunofluorescent (IF) staining demonstrated reduced percentage of positively labeled proliferating cells in aging BMSCs (Fig. S1D), indicating inhibited proliferative capacity. For the osteogenic function, alkaline phosphatase (ALP) and Alizarin red S staining assays were performed, and the results revealed decrease in osteogenesis of aging BMSCs (Fig. S1E–G). This phenomenon was confirmed by the Western blot analysis displaying downregulated expression of osteogenesis-related proteins in aging BMSCs, including Runt-related transcription factor 2 (RUNX2), ALP, and Osterix (OSX) (Fig. S1H). Moreover, aging BMSCs exhibited enhanced adipogenic capacity, as indicated by increased lipid droplet formation in oil red O staining after adipogenic induction (Fig. S1I). Cellular senescence was also evaluated using the senescence-associated beta-galactosidase (SA-β-gal) assay, which revealed a significant increase in the number of positively labeled senescent cells (Fig. S1J). These results confirm the dysregulated functional phenotype of senescent BMSCs and suggest correlations with the development of age-related osteoporosis.

**Fig. 1:**
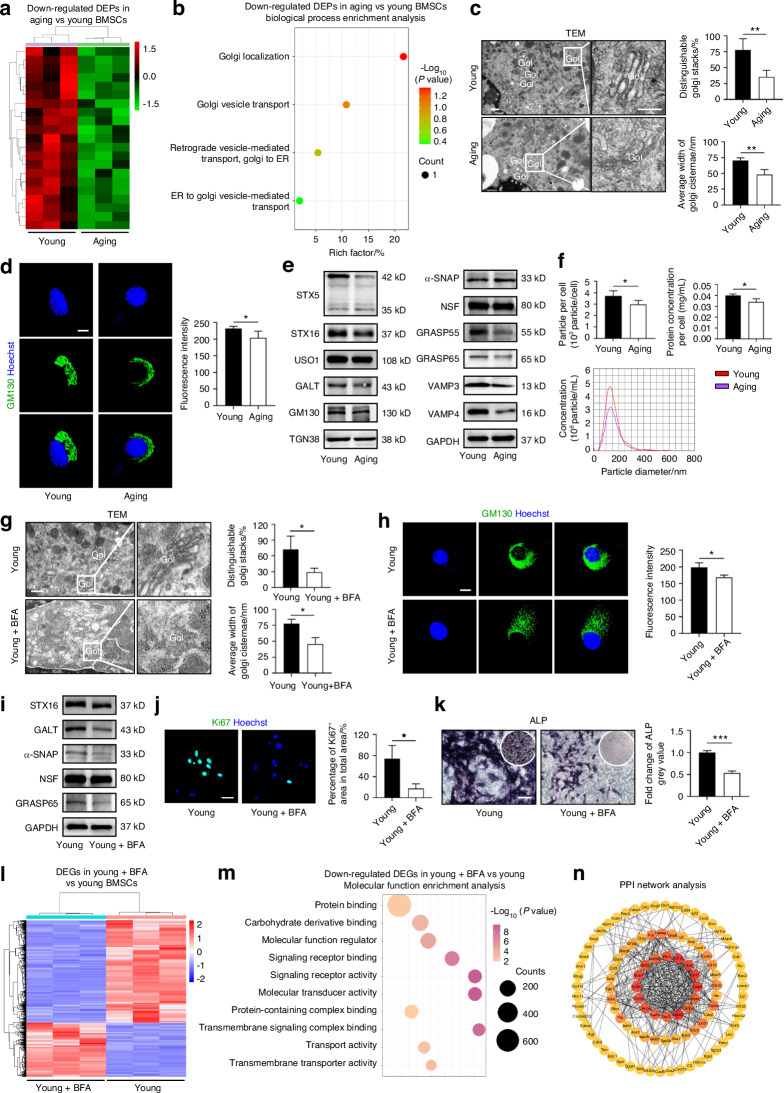
Golgi alterations contribute to functional decline of BMSCs in bone aging. **a** Hierarchical clustering of DEPs between aging BMSCs and young BMSCs, with protein abundance being Z-score normalized. **b** GO enrichment analysis of significantly downregulated DEPs in aging BMSCs. **c** TEM images showing Golgi ultrastructure in BMSCs and the quantification. Bar: 400 nm/250 nm. *n* = 4 per group. **d** BMSCs with GM130 IF staining (green) and nuclei (blue). Golgi fluorescence intensity was quantified. Bar: 10 μm. *n* = 4 per group. **e** Western blot analysis of Golgi vesicle trafficking-related protein expression in cultured BMSCs. **f** EV release function of the Golgi in BMSCs normalized to single cell by NTA and BCA protein quantification. *n* = 4 per group. **g** TEM images showing Golgi ultrastructure in BMSCs after BFA treatment and the quantification. Bar: 500 nm/250 nm. *n* = 3 per group. **h** BMSCs after BFA treatment with GM130 IF staining (green) and nuclei (blue). Golgi fluorescence intensity was quantified. Bar: 10 μm. *n* = 3 per group. **i** Western blot analysis of Golgi vesicle trafficking-related protein expression in BFA-treated BMSCs. **j** Ki67 IF staining to detect the proliferative capacity of BMSCs after BFA treatment and the quantification. Bar: 50 μm. *n* = 3 per group. **k** ALP staining of osteogenesis-induced BMSCs after BFA treatment and the quantification. Bars: 100 μm. *n* = 3 per group. **l** Hierarchical clustering of DEGs between BMSCs after BFA treatment and control, with gene abundance being Z-score normalized. **m** GO enrichment analysis of significantly downregulated DEGs on Molecular Function enrichment analysis in BMSCs after BFA treatment. **n** PPI network analysis of the DEGs related to the Molecular Function enrichment analysis in **m**. Data are presented as Mean ± SD. Statistical analyses were performed by Student’s *t* test. *P < 0.05; **P < 0.01; ***P < 0.001

Next, to determine the molecular basis for the altered function of BMSCs in aging, we performed proteomic analysis on BMSCs derived from aging and young mice using liquid chromatography coupled to tandem mass spectrometry (LC-MS/MS). A total of 122 differentially expressed proteins (DEPs) were identified (Fig. 1a; Table S1). Further analysis focused on significantly downregulated DEPs in aging BMSCs, and Gene Ontology (GO) enrichment analysis surprisingly revealed altered protein function closely related to Golgi function and vesicle transport (Fig. 1b). This result prompted us to investigate the potential structural and functional changes of the Golgi apparatus in aging BMSCs. Transmission electron microscopy (TEM) analysis demonstrated that the Golgi ultrastructure in young BMSCs existed in a relatively vital and full state, while the Golgi in aging BMSCs was in a remarkably wrinkle state, which was indicative of a putative declining function of vesicle secretion (Fig. 1c). IF staining using GM130, a cis-Golgi marker, confirmed the diminished Golgi in aging BMSCs (Fig. 1d). Golgi vesicle trafficking-related proteins, which are involved in the formation, movement, and fusion of vesicles, were further identified with significant downregulation in aging BMSCs (Fig. 1e). To directly investigate that whether the vesicular transport and secretion function was impaired in aging, we isolated EVs released from young and aging BMSCs and compared their quantity and protein content. Expectedly, nanoparticle tracking analysis (NTA) and BCA analysis revealed a decrease in both the number and protein amount of EVs released by aging BMSCs (Fig. 1f). Taken together, these findings indicate that BMSC senescence with structural and vesicular functional alterations of the Golgi apparatus occurs in bone aging.

To explore whether Golgi apparatus damage is a causative factor of cellular senescence, we treated young BMSCs with Brefeldin A (BFA), a Golgi disruptor.^24^ TEM and GM130 IF staining results revealed significant fragmentation of the Golgi apparatus in the BFA-treated group (Fig. 1g, h). Furthermore, Western blot analysis of expression of Golgi vesicle trafficking-related proteins after BFA treatment demonstrated that Golgi destruction markedly inhibited the secretory function of young BMSCs (Fig. 1i). To investigate the consequences of Golgi disruption on cellular function, we assessed the biological performance of BMSCs using Ki67 IF staining for proliferation and ALP staining for osteogenic potential (Fig. 1j, k). The findings indicated that the proliferative and osteogenic differentiation capacities of BMSCs were significantly diminished in BFA-treated group. Besides, to gain a deeper insight into the mechanisms by which Golgi damage induces a functional decline phenotype, we conducted RNA sequencing (RNA-seq) analysis after BFA treatment of BMSCs (Fig. 1l; Table S2). GO enrichment analysis indicated that multiple signaling pathways were affected by Golgi compromise, including “protein binding”, “signaling receptor binding”, and “transport activity”, suggesting broad mechanistic impacts (Fig. 1m). Protein-protein interaction (PPI) analysis additionally revealed a complex network of molecular targets with functional interactions after BFA treatment, in which several proliferation-related proteins (such as Cyclin A2, Ccna2) played a central role (Fig. 1n). Together, the above results provide evidence of the critical role Golgi plays in maintaining MSC homeostasis.

### Aging bone microenvironment induces BMSC dysfunction through compromised Golgi apparatus

**Fig. 2:**
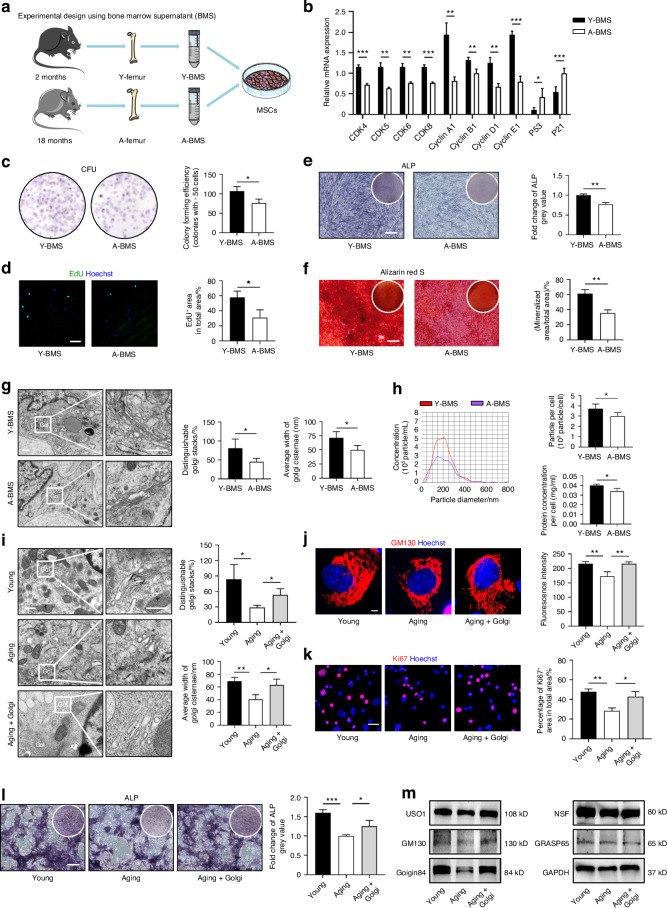
Aging bone microenvironment induces BMSC dysfunction through compromised Golgi apparatus. **a** Schematic diagram for the study design using BMS to treat BMSCs. Y-BMS, BMSC treated by young BMS. A-BMS, BMSC treated by aging BMS. **b** After 14 days of BMS treatment, qRT-PCR analysis of the mRNA expression levels of CDKs, Cyclins, P53 and P21. *n* = 3 per group. **c**, **d** Colony formation and EdU staining to detect the self-renewal capacity of BMSCs after BMS treatment and the quantification. Bar: 100 μm. *n* = 3 per group. **e**, **f** ALP and Alizarin red S staining of osteogenesis-induced BMSCs after BMS treatment and the quantification. Bars: 100 μm. *n* = 3 per group. **g** TEM images showing Golgi ultrastructure in BMSCs after BMS treatment and the quantification. Bar: 500 nm/250 nm. *n* = 4 per group. **h** EV release function of the Golgi in BMSCs normalized to single cell after BMS treatment by NTA and BCA protein quantification. *n* = 4 per group. **i** TEM images showing Golgi ultrastructure in BMSCs after Golgi fraction treatment and the quantification. Bar: 500 nm/250 nm. *n* = 3 per group. **j** BMSCs after Golgi fraction treatment with GM130 IF staining (red) and nuclei (blue). Golgi fluorescence intensity was quantified. Bar: 10 μm. *n* = 3 per group. **k** Ki67 IF staining to detect the proliferative capacity of BMSCs after Golgi fraction treatment and the quantification. Bar: 50 μm. *n* = 3 per group. **l** ALP staining of osteogenesis-induced BMSCs after Golgi fraction treatment and the quantification. Bars: 100 μm. *n* = 3 per group. **m** Western blot analysis of Golgi vesicle trafficking-related protein expression in Golgi fraction-treated BMSCs. Data are presented as Mean ± SD. Statistical analyses were performed by Student’s *t* test or one-way ANOVA. *P < 0.05; **P < 0.01; ***P < 0.001

A prominent characteristic of aging is the diseased microenvironment.^25^ Accordingly, we investigated the microenvironmental impact on Golgi and BMSC function in aging mice. We collected young and aging bone marrow supernatant (Y/A-BMS) by gradient centrifugation and treated cultured BMSCs with them (Fig. 2a). Quantitative real-time polymerase chain reaction (qRT-PCR) analysis revealed that the aging bone microenvironment significantly downregulated the expression of Cyclin-dependent kinase (CDK) genes and Cyclin genes in BMSCs (Fig. 2b). Notably, expression of cellular senescence markers, P21 and P53, was both significantly increased in the A-BMS group (Fig. 2b). Moreover, CFU assay and EdU staining demonstrated a significant decrease in the self-renewal capacity of BMSCs treated with A-BMS (Fig. 2c, d). Furthermore, following osteogenic induction, A-BMS-treated BMSCs showed significantly reduced ALP activity and formation of calcified nodules (Fig. 2e, f). We also examined the Golgi morphology and vesicular function of BMSCs after treated with BMS. TEM analysis revealed that the Golgi apparatus exhibited wrinkled states following A-BMS treatment (Fig. 2g), resembling the ultrastructural changes observed in BMSCs derived from aging mice. NTA and BCA assays further demonstrated that the aging bone microenvironment inhibited the EV release capacity of BMSCs (Fig. 2h). Collectively, the above findings suggest that aging bone microenvironment induces BMSC dysfunction with Golgi and vesicular alterations.

To further investigate that whether age-related functional decline of BMSCs can be retarded by restoring the Golgi apparatus, we isolated the Golgi fraction based on gradient centrifugation (Fig. S2A) and verified the enrichment of Golgi structural and functional proteins, rather than other organelle proteins, such as voltage-dependent anion channel 1 (VDAC1) for mitochondria (Fig. S2B). The isolated Golgi fraction was then used to treat BMSCs. TEM and GM130 IF staining results (Fig. 2i, j) revealed that aging BMSCs treated by Golgi fraction exhibited a relatively vital and complete state that closely resembled the Golgi structure in young BMSCs. In terms of functional assays, BMSCs in the Golgi treatment group showed significant improvements in both proliferation and osteogenic potential (Fig. 2k, l). This enhancement in biological function was accompanied by the restoration of several Golgi vesicle trafficking-related proteins, as indicated by Western blot analysis (Fig. 2m). These findings suggest that the restoration of Golgi function has the potential to rescue functional decline of aging BMSCs and improve their overall biological performance.

### Aging bone microenvironment disrupts the Golgi and impairs the regenerative capability of implanted MSC aggregates

**Fig. 3:**
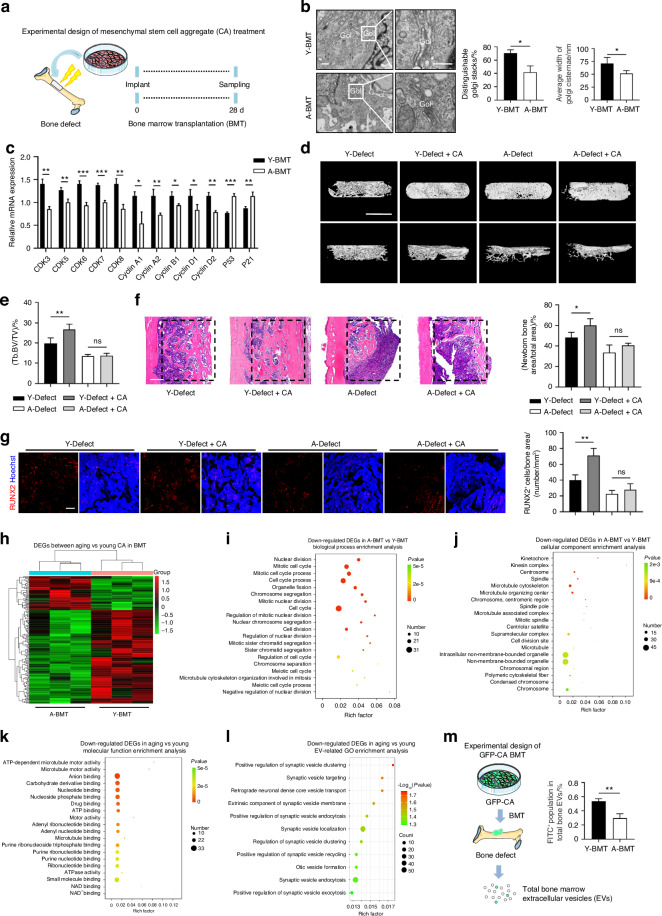
Aging bone microenvironment disrupts the Golgi and impairs the regenerative capability of implanted MSC aggregates. **a** Schematic diagram for the study design using a femoral defect model with BMT of CA. **b** TEM images showing Golgi ultrastructure in re-extracted CA after BMT and the quantification. Bar: 500 nm/250 nm. *n* = 4 per group. **c** After BMT, qRT-PCR analysis of the mRNA expression levels of cell cycle-related marker genes CDKs, Cyclins, P53 and P21 of re-extracted CA. *n* = 3 per group. **d**, **e** After 28 days of BMT with CA, micro-CT analysis of newborn bone mass and trabecular parameters at the femoral defect site. Bar: 1.5 mm. *n* = 4 per group. **f** After 28 days of BMT with CA, H&E staining for the femoral defect site and the quantification. Bar: 500 μm. *n* = 4 per group. **g** After 28 days of BMT with CA, RUNX2 IF staining (red) for the femoral defect site and quantification of the number of RUNX2^+^ cells in bone defect area. Bar: 100 μm, *n* = 4 per group. **h** Hierarchical clustering of DEGs in Y-BMT and A-BMT. Y-BMT, CA implanted into young bone defect. A-BMT, CA implanted into aging bone defect. **i**–**k** GO enrichment analysis of significantly down-regulated DEGs, categorized into Biological Process, Cellular Component, and Molecular Function. **l** The top enriched terms of down-regulated DEGs associated with EVs. **m** Schematic diagram for the study design of the whole bone marrow EV isolation and GFP^+^ EVs quantification. Data are presented as Mean ± SD. Statistical analyses were performed by Student’s *t* test or one-way ANOVA. *P < 0.05; **P < 0.01; ***P < 0.001; ns no significance

Resident MSCs help maintain tissue homeostasis and repair, while exogenous MSC transplantation has been widely used to promote tissue regeneration.^4^ The above findings inspired us to investigate that whether the aging bone microenvironment affects exogenously applied MSCs. In this regard, we utilized CA established by SHED, the proved readily available MSC source for translational transplantation and regenerative therapies possessing similar characteristics with BMSCs.^15,26^ After confirming their MSC property and identity (Fig. S3A–G), SHED were induced for CA, which developed a thickness and dense structure (Fig. S3H) with a mesh-like arrangement of tightly connected cells under light microscope (Fig. S3I) and dense collagen fibers under scanning electron microscopy (SEM) (Fig. S3J). Histological analysis using Hematoxylin and eosin (H&E) and Masson’s trichrome staining confirmed the presence of a substantial amount of ECM within the CA (Fig. S3K,S3L). To investigate the impact of the aging bone microenvironment on implanted MSCs in vivo, we established a bone defect experimental model with bone marrow transplantation (BMT) of CA into the defect site of aging and young mice (A/Y-BMT) (Fig. 3a). After transplantation for 72 h, the implanted CA was extracted and examined. TEM analysis revealed a wrinkled Golgi structure in the A-BMT group (Fig. 3b), similar to the observations in BMSCs derived from aging mice or treated with A-BMS. Furthermore, qRT-PCR analysis showed significant downregulation of Cyclin genes and CDK genes in CA implanted into aging bone microenvironment, suggesting senescence-related cell cycle arrest (Fig. 3c). In addition, the expression of P21 and P53 was significantly enhanced in the A-BMT group (Fig. 3c). To assess the functional results of CA after BMT, we extended the implantation time and evaluated therapeutic effects on femoral defects after 28 days of treatment. Micro-CT and quantitative analysis demonstrated that CA treatment successfully promoted bone repair in young mice (Fig. 3d, e). However, in aging mice, CA treatment did not result in a significant increase in the bone mass at the femoral defects, indicating impaired regenerative efficacy (Fig. 3d, e). Histological analysis of the bone defect area using H&E staining confirmed increased trabecular bones in young mice by CA compared to the non-transplanted control group, while no significant difference was observed in aging mice (Fig. 3f). After 28 days of treatment, IF staining for RUNX2 further revealed effective acceleration of osteogenesis by CA in only young but not aging mice (Fig. 3g). Collectively, these results suggest that aging bone microenvironment disrupts the Golgi and impairs the regenerative capability of implanted MSC aggregates.

Next, we investigated whether the aging bone microenvironment affects the gene expression profile of CA. After implantation of CA in aging and young mice for 72 h, CA was extracted and underwent RNA-seq analysis (Fig. 3h; Table S3). The downregulated differentially expressed genes (DEGs) were further annotated to three GO categories (Fig. 3i–k). Notably, both the “Biological Process” and the “Cellular Component” categories were significantly enriched for DEGs related to cell division, including “cell cycle”, “cell cycle process”, “chromosome”, “chromosome segregation”, and “cell division” (Fig. 3i, j). Surprisingly, GO enrichment analysis further revealed that EV transport and release were significantly enriched in the downregulated DEGs, including “synaptic vesicle targeting”, “retrograde neuronal dense core vesicle transport”, “positive regulation of synaptic vesicle recycling”, and “positive regulation of synaptic vesicle exocytosis” (Fig. 3l). These results suggest that the aging bone microenvironment inhibits the regenerative effects of CA with attenuating or impairing the EV release function of the Golgi apparatus.

To further test the hypothesis that the aging microenvironment suppresses CA function with inhibiting EV release, we again performed in vivo implantation experiments. Firstly, we constructed SHED expressing the green fluorescent protein (GFP), induced them to establish GFP-CA, and isolated GFP-CA-EVs. Flow cytometric analysis revealed that approximately 29.5% of the EV population was GFP-positive (Fig. S3M, N). Subsequently, we implanted GFP-CA into the bone defects of young and aging mice, respectively, and then collected whole bone marrow EVs to analyze the proportion of GFP-positive EVs within (Fig. 3m). The results demonstrated that the proportion of GFP-positive EV population in the whole bone marrow of young mice was higher than that in aging mice (Fig. 3m), which was statistically significant. Together, these findings provide further evidence that the aging bone microenvironment inhibits the EV secretion capacity of the implanted CA.

### Nanoscale profiling of CA-EVs identifies regenerative and Golgi regulatory property

**Fig. 4:**
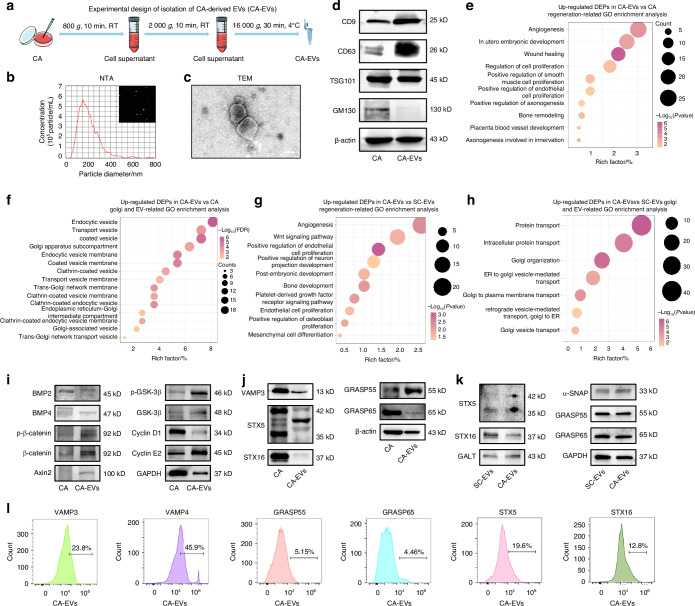
Nanoscale profiling of CA-EVs identifies regenerative and Golgi regulatory property. **a** Schematic diagram for the isolation process of CA-EVs. **b** NTA showing the size distribution of CA-EVs. **c** Representative TEM image showing the morphology of CA-EVs. Bar: 100 μm. **d** Western blot analysis of the EV marker expression. **e** The top enriched terms of up-regulated DEPs between CA-EVs and CA associated with regeneration. **f** The top enriched terms of up-regulated DEPs between CA-EVs and CA associated with Golgi and EVs. **g** The top enriched terms of up-regulated DEPs between CA-EVs and SC-EVs associated with regeneration. **h** The top enriched terms of up-regulated DEPs between CA-EVs and SC-EVs associated with Golgi and EVs. **i** Western blot analysis of development/regeneration-related protein expression in CA and CA-EVs. **j** Western blot analysis of the Golgi regulatory protein expression in CA and CA-EVs. **k** Western blot analysis of the Golgi regulatory protein expression in SC-EVs and CA-EVs. **l** Flow cytometric analysis of Golgi regulatory surface protein expression in CA-EVs

Inspired by the above findings, we assumed that replenishment of EVs might be beneficial for MSC function in aging. We isolated CA-EVs as previously described and characterized them at the nanoscale level according to the International Society of Extracellular Vesicles (ISEV2023) guideline^27^ (Fig. 4a–d). We further characterized the specific proteomic features of CA-EVs by LC-MS/MS, identifying a total of 2398 total proteins compared to CA (Fig. S4A; Table S4). Intriguingly, we identified that the DEPs up-regulated in CA-EVs compared to their parental CA were significantly enriched in development- and regeneration-related signaling pathways (Fig. 4e), such as “in utero embryonic development”, “wound healing”, “regulation of cell proliferation”, and “bone remodeling”. GO enrichment analysis further revealed that CA-EVs exhibited increased expression of Golgi function-related proteins than CA, particularly secretory function proteins, such as “transport vesicle”, “trans-Golgi network membrane”, “Golgi-associated vesicle”, and “trans-Golgi network transport vesicle” (Fig. 4f). Moreover, we analyzed and compared the protein profiles of CA-EVs with single-cell MSC-derived EVs (SC-EVs) (Fig. S4B; Table S5). GO enrichment analysis again revealed that CA-EVs upregulated DEPs over SC-EVs associated with development- and regeneration-related pathways, including “Wnt signaling pathway”, “bone development”, and “mesenchymal cell differentiation” (Fig. 4g). Additionally, GO enrichment analysis indicated that CA-EVs upregulated DEPs over SC-EVs related to protein transport, Golgi organization, and Golgi vesicle trafficking (Fig. 4h).

Westen blot analyses were subsequently used to validate the above findings. Results confirmed the expression of development- and regeneration-related signaling proteins in CA-EVs, including those of the bone morphogenetic proteins (BMPs), Wnt pathway regulators, and Cyclins (Fig. 4i). Furthermore, Golgi vesicle trafficking-related proteins in CA-EVs were verified (Fig. 4j, k). Notably, the Golgi assembly protein STX5, as well as other molecules, showed higher expression levels in CA-EVs than SC-EVs (Fig. 4k). We have previously discovered that MSC-derived EVs promote Golgi assembly through surface soluble *N*-ethylmaleimide-sensitive factor attachment protein receptor (SNARE) proteins.^24^ Accordingly, we analyzed surface profiles of CA-EVs using flow cytometry and discovered that multiple Golgi regulatory proteins were exposed, including vesicle-associated membrane protein 3 (VAMP3) and VAMP4 for endosomal fusion with Golgi,^28,29^ the Golgi reassembly and stacking protein 55 (GRASP55) and GRASP65,^30,31^ and STX5 and STX16 for Golgi vesicular transport^32,33^ (Fig. 4l). Collectively, these results suggest that CA-EVs possess regenerative and Golgi regulatory property and may play a crucial role in restoring Golgi in target cells.

### CA-EV treatment restores Golgi homeostasis and BMSC function *via* STX5

**Fig. 5:**
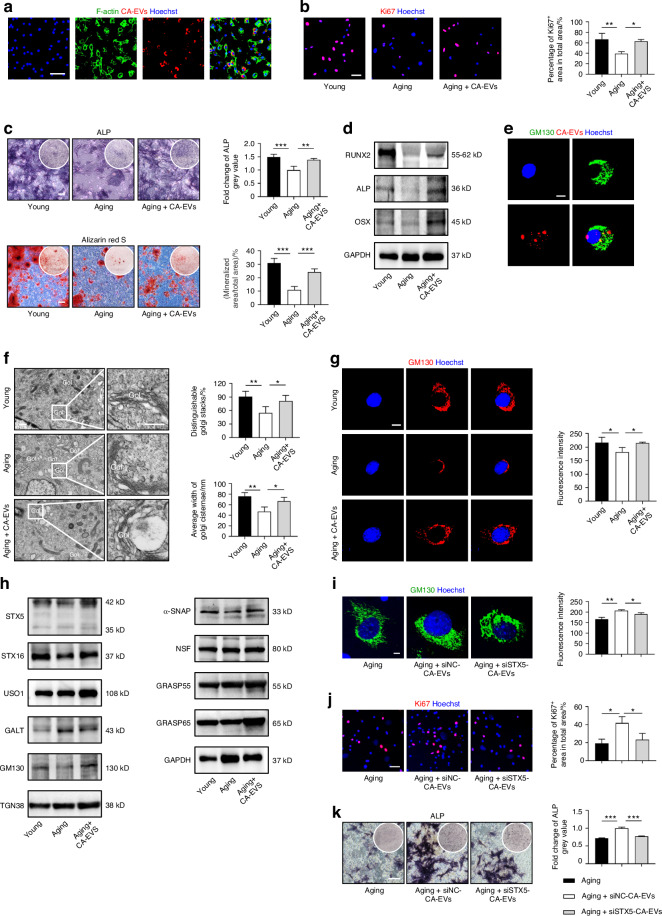
CA-EV treatment restores Golgi homeostasis and BMSC function *via* STX5. **a** CA-EVs phagocytosed by BMSCs in vitro. Bar: 50 μm. **b** Ki67 IF staining to detect the proliferative capacity of BMSCs after CA-EV treatment and the quantification. Bar: 50 μm. *n* = 3 per group. **c** ALP and Alizarin red S staining of osteogenesis-induced BMSCs after CA-EV treatment and the quantification. Bars: 100 μm. *n* = 4 per group. **d** Western blot analysis of osteogenesis-related protein expression in BMSCs after CA-EV treatment. **e** IF staining of BMSCs with GM130 for Golgi apparatus (green), internalized CA-EVs (red) and nuclei (blue). Bar: 10 μm. **f** TEM images showing Golgi structure in BMSCs after CA-EV treatment and the quantification. Bar: 250 nm. *n* = 4 per group. **g** BMSCs with GM130 IF staining (red) for Golgi and nuclei (blue). Golgi fluorescence intensity was quantified. Bar: 10 μm. *n* = 4 per group. **h** Western blot analysis of Golgi regulatory and vesicle trafficking-related protein expression in cultured BMSCs after CA-EV treatment. **i** BMSCs with GM130 IF staining (green) and nuclei (blue). Golgi fluorescence intensity was quantified. Bar: 10 μm. *n* = 3 per group. siNC-CA-EVs, CA-EVs derived from MSCs transfected with siRNA-negative control; siSTX5-CA-EVs, CA-EVs derived from MSCs transfected with siRNA-STX5. **j** Ki67 IF staining to detect the proliferative capacity of BMSCs and the quantification. Bar: 50 μm. *n* = 3 per group. **k** ALP staining of osteogenesis-induced BMSCs and the quantification. Bars: 100 μm. *n* = 3 per group. Data are presented as Mean ± SD. Statistical analyses were performed by one-way ANOVA. *P < 0.05; **P < 0.01; ***P < 0.001

Next, we investigated whether CA-EVs improve the function and Golgi status of aging BMSCs. We isolated BMSCs from 18-month-old aging mice and treated them with CA-EVs ex vivo. As expected, fluorescent staining showed that CA-EVs were phagocytosed by BMSCs (Fig. 5a). Ki67 IF staining demonstrated that CA-EV treatment significantly promoted the proliferation of aging BMSCs (Fig. 5b). Furthermore, CA-EV treatment enhanced the osteogenic differentiation of aging BMSCs, as evidenced by in ALP and Alizarin red staining (Fig. 5c). Western blot analysis confirmed that the expression of osteogenic proteins, RUNX2, ALP and OSX, was upregulated in the CA-EV treatment group (Fig. 5d). For the Golgi apparatus, intriguingly, GM130 IF staining revealed that PKН26-labeled CA-EVs co-localized with recipient Golgi (Fig. 5e). Subsequent TEM and GM130 IF staining analysis collectively demonstrated that CA-EV treatment restored the Golgi structure in aging BMSCs, shifting it from a wrinkled state to a vital and complete state that closely resembled the Golgi structure in young BMSCs (Fig. 5f, g). Moreover, Western blot analysis exhibited that expression of Golgi vesicle trafficking-related proteins in aging BMSCs was promoted by CA-EV treatment, suggesting enhanced function in vesicle synthesis and transport (Fig. 5h). Together, these findings highlight that CA-EV treatment restores Golgi structure and function, which contribute to promoting the proliferation and osteogenic differentiation of aging BMSCs.

To elucidate the mechanism by which CA-EVs restore Golgi function, we focused on Golgi vesicle trafficking-related proteins highly expressed in CA-EVs than SC-EVs. Accordingly, we selected the STX5 protein. We constructed and tested small interfering RNAs (siRNAs) for knocking down STX5 in SHED (Fig. S5A), and confirmed using Western blot experiments to show significant inhibition of STX5 protein expression in both CA and CA-EVs (Fig. S5B). IF staining revealed that siSTX5-CA-EVs were effectively internalized by BMSCs (Fig. S5C). We then isolated siSTX5-CA-EVs and treated aging BMSCs. GM130 IF staining revealed that the Golgi recovery effect of CA-EVs was diminished in the siSTX5-CA-EVs group, with only minimal improvement observed, suggesting STX5 be a crucial protein molecule for CA-EVs to exert their function (Fig. 5i). Ki67 IF staining and ALP staining further demonstrated that knockdown STX5 protein in CA-EVs significantly inhibited their ability to promote the biological function of BMSCs (Fig. 5j, k). Collectively, our experimental data dissect the molecular mechanism by which CA-EVs promote Golgi function in aging BMSCs through the exposing of STX5.

### CA-EV transplantation promotes aging bone regeneration

**Fig. 6:**
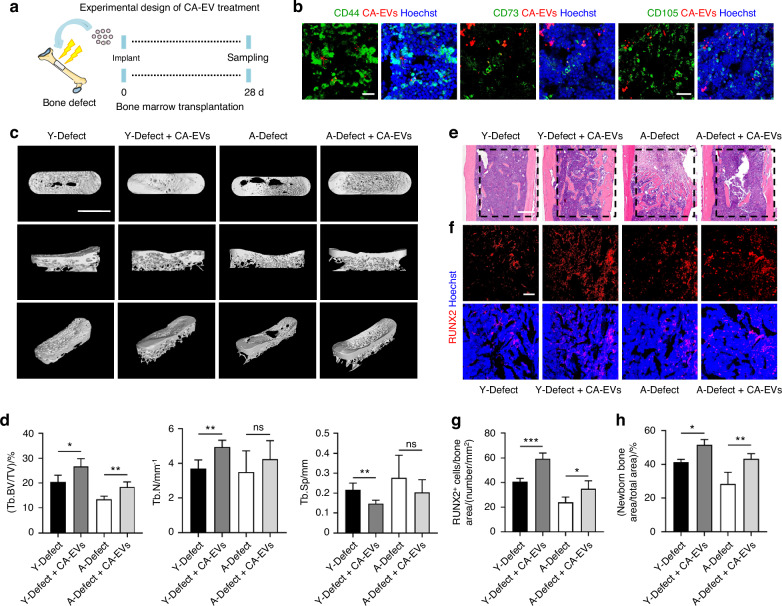
CA-EV transplantation promotes aging bone regeneration. **a** Schematic diagram for the study design using a femoral defect model and CA-EV transplantation. **b** Local applied CA-EVs were phagocytosed by BMSCs in vivo. Bar: 20 μm. **c**, **d** After 28 days of treatment with CA-EVs, micro-CT analysis of newborn bone mass and trabecular parameters at the femoral defect site. Bar: 1.5 mm. *n* = 4 per group. **e** After 28 days of treatment with CA-EVs, H&E staining for the femoral defect site. Bar: 500 μm. **f** After 28 days of treatment with CA-EVs, RUNX2 IF staining (red) for the femoral defect site. Bar: 100 μm. **g** The quantification of the number of RUNX2^+^ cells in bone defect area. *n* = 4 per group. **h** The quantification of the newborn bone area in H&E staining. *n* = 4 per group. Data are presented as Mean ± SD. Statistical analyses were performed by one-way ANOVA. *P < 0.05; **P < 0.01; ***P < 0.001; ns no significance

The above results encouraged us to further evaluate the potential of using CA-EVs to enhance the bone regenerative capacity in aging mice. To this end, we established a CA-EV transplantation model in aging bone defect and investigated its efficacy (Fig. 6a). After local delivery of CA-EVs to femoral defects for 48 h, IF staining revealed that CA-EVs were effectively internalized by CD44-, CD73-, and CD105-labeled resident BMSCs (Fig. 6b). Importantly, after 28 d of CA-EV administration, micro-CT analysis and quantitative measurements demonstrated that CA-EVs significantly promoted bone regeneration at the femoral defects in both young and aging mice, as evidenced by increased Tb.BV/TV (Fig. 6c, d). Furthermore, H&E and RUNX2 IF staining collectively discovered increased trabecular bone formation and improved in vivo osteogenesis after CA-EV treatment, either in young or in aging mice (Fig. 6e–h). Taken together, these findings indicate that CA-EV transplantation promotes aging bone regeneration, which serves as an optimized therapeutic approach in aging microenvironment than CA transplantation.

### CA-EVs empower senescent BMSCs and alleviate age-related bone loss

**Fig. 7:**
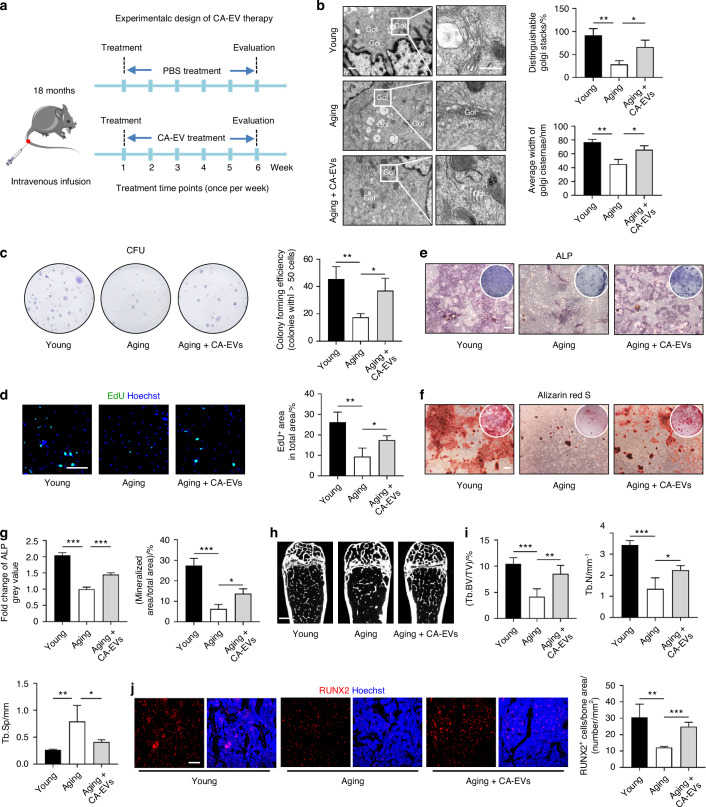
CA-EVs empower senescent BMSCs and alleviate age-related bone loss. **a** Schematic diagram for the study design using intravenous infusion of CA-EVs. The infusion was performed in mice started at 18-month-old of age once per week for a total of 6 weeks. **b** TEM images showing Golgi ultrastructure in BMSCs after CA-EV infusion and the quantification. Bar: 400 nm/250 nm. *n* = 4 per group. **c**, **d** Colony formation and EdU staining to detect the self-renewal capacity of BMSCs after CA-EV infusion and the quantification. Bar: 100 μm. *n* = 4 per group. **e**–**g** ALP and Alizarin red S staining of osteogenesis-induced BMSCs after CA-EV infusion and the quantification. Bars: 100 μm. *n* = 3 per group. **h**, **i** After 6 weeks of treatment with CA-EVs, micro-CT analysis of femoral bone mass and trabecular parameters for the femoral bone. Bar: 250 μm. *n* = 4 per group. **j** After 6 weeks of treatment with CA-EVs, RUNX2 IF staining (red) for the femoral bone and the quantification of the number of RUNX2^+^ cells in bone defect area. Bar: 100 μm. *n* = 4 per group. Data are presented as Mean ± SD. Statistical analyses were performed by one-way ANOVA. *P < 0.05; **P < 0.01; ***P < 0.001

Finally, we explored the potential of using CA-EVs to promote function of endogenous senescent BMSCs and prevent age-related bone loss. We established a chronic intermittent intravenous infusion protocol for systemic delivery of CA-EVs into 18-month-old aging mice at 1-week intervals (Fig. 7a). To monitor the biodistribution of CA-EVs in vivo, DIR-labeled CA-EVs were infused, and 24 h after intravenous infusion, CA-EVs predominantly engrafted in the liver and the spleen, while also being detected in the femur and the tibia (Fig. S6A). Fluorescent imaging confirmed the presence of PKH26-labeled CA-EVs in the femur (Fig. S6B). After 6 consecutive weeks of treatment, the mice were euthanized, and BMSCs were isolated and analyzed ex vivo. TEM results revealed that while aging BMSCs maintained a wrinkled state compared to young BMSCs, their Golgi structure exhibited significant improvement and partial restoration to an intact condition following the intravenous infusion of CA-EVs (Fig. 7b). Expectedly, CA-EV infusion enhanced the self-renewal capacity of aging BMSCs, as demonstrated by CFU and EdU assays (Fig. 7c, d). Furthermore, ALP and Alizarin red S staining revealed that CA-EV infusion significantly promoted osteogenesis of aging BMSCs (Fig. 7e–g). Importantly, after 6 weeks of treatment, micro-CT analysis demonstrated that CA-EV infusion substantially mitigated age-related osteoporosis, as evidenced by an increase in the trabecular bone volume and number in aging mice (Fig. 7h, i). Moreover, RUNX2 IF staining results showed that CA-EV infusion enhanced osteogenesis in aging mice (Fig. 7j), with suppressed osteoclastic bone resorption shown by Tartrate-resistant acid phosphatase (TRAP) staining (Fig. S6C). These results provide further evidence that the infusion of CA-EVs facilitates a balanced shift between osteogenesis and osteoclastogenesis, thereby maintaining bone homeostasis in aging mice. Collectively, these findings suggest that CA-EVs empower senescent BMSCs and alleviate age-related bone loss.

In brief summary, we discovered that aging bone microenvironment impaired the Golgi apparatus and diminished BMSC function and regeneration. Replenishment of CA-EVs rescued Golgi dysfunction and empowered senescent BMSCs through exposing the Golgi regulatory protein STX5. Importantly, in vivo administration of CA-EVs significantly enhanced bone regeneration and improved bone mass in aging mice (Fig. S7). These findings provide insights into Golgi regulation in stem cell senescence and bone aging, which also suggest the therapeutic value of CA-EVs for treating age-related osteoporosis and promoting aging bone regeneration.

## Discussion

Healthy aging is among the major focuses of scientific research in the medical field.^34,35^ While certain species can replenish their stem cell pools, vertebrates inevitably undergo aging due to the age-related decline of tissue-resident stem cells and their niches, leading to the development of degenerative diseases.^36,37^ Bone aging, manifested by osteoporosis and reduced bone regenerative capacity, is the most prevalent age-related skeletal alteration, yet there are currently no established therapeutic strategies specifically targeting this process. In this study, we demonstrate that impaired Golgi structure and function, resulting in dysregulated EV release, played a significant role in the BMSC dysfunction in the aging microenvironment. Notably, exogenous MSC aggregates implanted in the aging bone were similarly affected by the senescent microenvironment, leading to diminished regenerative capacity. Importantly, supplementation with CA-EVs, rather than CA themselves, restored Golgi function, which in turn empowered senescent endogenous BMSCs, promoted the regeneration of aging bone defects, and effectively alleviated the age-related osteoporotic phenotype. Thus, our study unveils a novel organelle mechanism underlying stem cell functional impairment in the aging microenvironment and proposes a corresponding therapeutic strategy to address this issue. Further exploration of interventions aiming at promoting aging bone regeneration by improving the senescent microenvironment and rescuing resident stem cells will hold promise for the treatment of age-related bone disease.

With the global population aging and increasing life expectancy, according to the World Health Organization (WHO), the number of individuals over the age of 60 is projected to reach 2.1 billion worldwide by 2050. Age-related bone disease, such as osteoporosis, will become a significant and growing public health concern, with an estimated 400 million people aged 50 and older affected worldwide by 2050.^38,39^ Regrettably, the precise pathogenesis of bone aging remains poorly understood, and effective therapeutic interventions are still lacking. Therefore, it is crucial to explore novel therapeutic targets to develop more efficacious treatments for bone aging.^40^ In this study, we identified the reduced synthesis and release of EVs as a key factor contributing to BMSC dysfunction in the aging microenvironment, which is attributed to impaired Golgi, consistent with previous research indicating EVs be an important regulator of MSC function.^41–43^ It is well documented that the aging microenvironment is characterized with a pro-inflammatory profile,^44–46^ but the detailed niche factor regulating aging BMSCs will require further analysis. Despite the niche influence, interestingly, our results further reveal that CA-EVs expressed Golgi functional proteins, suggesting their potential to restore impaired Golgi structure upon replenishment. Indeed, the functional cargos within stem cell-derived EVs have been reported to strongly regulate cell function. These, for an example, include the ability of stem cell-derived EVs to modulate immune cells, which holds promise for alleviating inflammation.^18^ In the aging research, stem cell-derived EVs have been reported to transfer proliferating cell nuclear antigen (PCNA) and a cocktail of protein components in a synergistic way to induce the activation of several canonical signaling pathways, such as Wnt and Sirtuin signaling, to rejuvenate aged BMSCs and slow age-related degeneration.^10,47^ Here, internalization of CA-EVs by senescent BMSCs significantly enhanced their proliferative and osteogenic differentiation capabilities, ultimately leading to the amelioration of bone aging, at least in part through the recovery of Golgi function. Notably, while the phenotype of low bone turnover may be observed in aged mice,^48,49^ a recent study has reported increased osteoclastic bone resorption as a sign of hematopoietic aging due to niche action of senescent skeletal stem cells,^50^ which can be attributed to the senescence-associated secretory phenotype (SASP) to promote survival of monocyte osteoclast progenitors.^51^ Here, CA-EV infusion also suppressed osteoclastic bone resorption in aging mice, whereas whether CA-EVs directly regulate osteoclastogenesis or indirectly through alleviating resident MSC senescence is still elusive. Thus, our findings provide a novel target for the development of anti-bone aging therapeutics for clinical application.

In recent years, regenerative medicine, with its focus on repairing damaged tissues through stem cell transplantation and/or mobilization, has demonstrated promising application prospects.^52–54^ It has been increasingly recognized that stem cell function is tightly regulated by the surrounding niche.^25,55^ CA, constructed using a stem cell self-assembly approach, has been proposed to mimic progenitor cell condensation, which serves as generative centers for organogenesis and determinants of subsequent tissue formation and developmental patterns.^12,13^ The ECM within CA is believed to provide an organized microenvironment for stem cells to function, and the regenerative potential of CA has been demonstrated in numerous experimental models.^13,56^ Particularly, our clinical trials have demonstrated the critical role of CA in directing the formation and regeneration of dental tissues, highlighting their translational significance.^15,16^ EVs play a crucial role in mediating intercellular communication and have gained increasing attention as potential therapeutic or drug delivery agents due to their ability to transport bioactive molecules and overcome biological barriers.^57–62^ Our previous research has identified differences in odontogenic, osteogenic, and angiogenic properties between CA-EVs and their parental CA, suggesting enhanced biological function.^14,20^ Notably, CA-EVs also possess advantages over SC-EVs, at least for MSCs, which have higher yield and elevated expression of functional cargoes, as shown here and in our previous studies.^14,20^ In this study, we further demonstrate that CA-EVs expressed Golgi regulatory proteins, co-localized with Golgi upon cellular entry, and significantly improved Golgi structure and function. Both in vitro and ex vivo experiments revealed that CA-EV treatment enhanced the proliferation and osteogenic differentiation of aging BMSCs, which contributed to therapeutic effects of CA-EVs to promote regeneration of aging bone defects and alleviate osteoporosis in aging mice. Further studies are warranted to improve the bone-targeting properties and utilization of CA-EVs.^63,64^ While the precise mechanisms by which CA-EVs retard senescence remain to be elucidated, our findings suggest the involvement of Golgi as a novel target for bone aging and indicate the potential of using EV replenishment to restore Golgi structure and function, thereby enhancing the biological function of senescent BMSCs.

The Golgi apparatus is essential for vesicular secretion, as it provides a platform for the processing, sorting and packaging of cargos into vesicles and regulates vesicle release through a series of proteins.^65^ Notably, our recent study has revealed that in a particular EV-deficient mouse model, the Golgi apparatus exhibits a fragmented and dysfunctional state. Conversely, treatment with exogenous MSC-derived EVs preserves liver homeostasis and regeneration by regulating the formation and function of Golgi.^24^ This study uncovers the significant paradigm that EV administration rescues Golgi deficiencies, suggesting its potential utility as a therapeutic approach for related disease treatment.^24,66^ Additional research has implicated the Golgi apparatus in tumor migration and invasion during carcinogenesis, the pathogenesis and progression of neurodegenerative disorders, and the regulation of plant responses to environmental stressors.^67,68^ However, the multifaceted role of the Golgi apparatus in vertebrate cell senescence remains poorly understood. It has been reported that structural alterations of Golgi are often accompanied by changes in function.^69^ For example, in secretory cells, Golgi usually has a well-developed structure to fulfill the need to secrete large amounts of proteins, while in non-secretory cells, Golgi may exhibit a smaller structure to adapt to the normal function of the cell. In this study, we demonstrate that in aging stem cells, the Golgi apparatus underwent significant structural wrinkling alterations, accompanied by a decline in EV release. Furthermore, chemical Golgi disruption induces functional decline of BMSCs, and Golgi replenishment contributes to rescued function of aging BMSCs. Transcriptomic analysis indicates that Golgi homeostasis regulates multiple signaling in BMSCs, whereas how indeed the impaired Golgi apparatus affects BMSCs function and senescence will be determined in the future. Importantly, treatment of aging BMSCs with CA-EVs significantly improved Golgi structure with a marked enhancement in EV release. These findings suggest that organelles like the Golgi apparatus critically contribute to the cellular senescence process. Mechanistically, we have previously reported that EVs use surface VAMP3 to assemble with recipient Golgi.^24^ In this study, we further confirm that multiple Golgi regulatory proteins were exposed on the surface of CA-EVs, among which STX5 is a particular donor molecule mediating CA effects on recipient Golgi, suggesting membrane-signal related assembly mechanisms. The detailed molecular function will be explored in our subsequent studies.

In summary, CA-EV replenishment rescues Golgi dysfunction and empowers senescent BMSCs in the aging bone microenvironment, which enhances aging bone defect recovery and improves bone mass. Our findings provide insights into Golgi regulation in stem cell senescence and bone aging, suggesting the therapeutic value of CA-EVs for treating age-related osteoporosis and promoting aging bone regeneration.

## Materials and methods

### Animals

2-month-old and 18-month-old male C57BL/6J mice were purchased from Beijing Viton Lever, China, and maintained in a controlled environment with a temperature of 24 ± 2 °C, relative humidity of (55 ± 5)%, and a 12 h light/dark cycle with free access to water and standard chow. All animal experimental protocols were approved by the Animal Ethics Committee of The Fourth Military Medical University and were conducted in accordance with the Guidelines of Intramural Animal Use and Care Committee of The Fourth Military Medical University.

### Construction and morphological observation of CA

The process of constructing CA was performed following our previous studies. Briefly, the 4th-passaged SHED was inoculated in a 6-well culture plate at a density of 1 × 10^6^/well. When the cells reached 90% confluency, the medium was changed to the cell aggregate-induction culture media: α-MEM medium (Gibco, USA) supplemented with 10% FBS (ExCell Bio, China) and 50 μg/mL Vitamin C (Sigma-Aldrich, USA) under direct light-avoidance conditions. The medium was changed every 2 days, and after 10 d of induction, CA was observed under the microscope with thick edges separated from the dish bottom. For SEM observation, CA was washed with PBS and fixed with 4% paraformaldehyde (PFA, Coolaber, China) for 8 h at 4 °C. After washing with PBS, CA was sequentially passed through concentration ethanol (FUYU, China) of 30%, 50%, 70%, 80%, 90% and 100%, with 5 min for each concentration. CA was then immersed in hexamethyldisilazane (Sigma-Aldrich, USA) for 30 min at room temperature and dried naturally. The dried CA was stuck on the sample table and observed under the SEM.

### Isolation, characterization and application of CA-EVs

CA-EVs were isolated from the cultured medium of CA, as described previously. Briefly, established CA was cultured in α-MEM medium with 10% EV-free FBS for 48 h. The supernatant was centrifuged at 800 × *g* for 10 min to remove cells, centrifuged at 2 000 × *g* for 10 min to remove cell debris, and further centrifuged at 16 000 × *g* for 30 min to obtain CA-EVs. The particle number of CA-EVs was detected using NTA and the protein content was quantified using the BCA method normalized to the single cell (Beyotime, China). TEM was used to observe the morphology of CA-EVs, and Western blot analysis was used to detect the protein expression in CA-EVs. For the treatment of BMSCs in vitro, CA-EVs were added with a protein concentration of 150 μg/mL. For intravenous infusion in vivo, CA-EVs were prepared using sterile PBS with a protein concentration of 300 μg/mL and were injected into the caudal vein of mice at 200 μL (60 μg), weekly injections for 6 weeks.

### Statistical analysis

All the experimental data were presented as Mean ± standard deviation (SD). Statistical analysis was performed using GraphPad Prism 8.0. Significance was assessed by Student’s *t* test for two group comparisons, and one-way ANOVA followed by the Turkey’s post-hoc test for multiple group comparisons. Values of *P* < 0.05 were considered statistically significant.

## Supplementary information

Supplementary Information

Table S1

Table S2

Table S3

Table S4

Table S5

Table S6

## Data Availability

All data included in this study are available upon reasonable request of corresponding authors.
