# Supplementary material for: Golgi-restored vesicular replenishment retards bone aging and empowers aging bone regeneration
Source: Bone Res. 2025 Feb 8;13:21. doi: 10.1038/s41413-024-00386-w (PMC11807224; doi:10.1038/s41413-024-00386-w)
Supplement: Supplementary file 1 — Supplementary Information [file 41413_2024_386_MOESM1_ESM.docx]

**Supplementary Materials and Methods**

**Isolation and culture of MSCs**

Human exfoliated deciduous tooth samples were obtained from School of Stomatology, The Fourth Military Medical University with informed consents and ethics approval. SHED culture was performed as described previously. Briefly, pulp tissue from the deciduous teeth was rinsed 3 times with PBS (Servicebio, China) and cut into pieces, and type I collagenase (Sigma-Aldrich, USA) was added for digestion for 2 h at 37°C. Then, α-MEM medium (Gibco, USA) supplemented with 10% fetal bovine serum (FBS, ExCell Bio, China) was added to terminate digestion and was centrifuged at 500 g for 5 min. The tissue was resuspended and cultured in an atmosphere of 5% CO_2_ at 37°C. The fresh medium was changed every 3 days, and after 7 days the passages were processed.

The culture of murine BMSCs was performed as previously described. Briefly, mouse femora and tibiae were isolated on an ultra-clean bench. The bone marrow cavity was repeatedly rinsed using PBS containing 2% FBS, centrifuged at 500 g for 10 min, added with α-MEM medium (Gibco, USA) supplemented with 20% FBS (ExCell Bio, China), 100 U/ml penicillin (Gibco, USA) and 100 g/ml streptomycin (Gibco, USA) to resuspend the cell precipitates, and were seeded into 10 cm culture dishes. After 24 h in an atmosphere of 5% CO_2_ at 37°C, cells were added with a fresh medium after PBS wash, and the medium was changed every 2 days. After 14 days, BMSCs were passed by treatment with trypsin (Gibco, USA). For BFA treatment, BMSCs were treated with 0.5 mM BFA (Invitrogen, USA).

**Cell functional experiment**

Colony-forming assay: For colony formation, primary MSCs were seeded into 5 cm culture dishes at a density of 1x10^3^ cells/dish, and the culture medium was changed every 3 days. After 14 days, MSCs were fixed with 4% PFA and stained with crystal violet (Sigma-Aldrich, USA) at a mass fraction of 0.1%.

EdU assay: EdU assay was performed using the kFluor488 Click-iT EdU assay kit (KeyGEN BioTECH, China). Briefly, the 3rd-passaged MSCs were seeded on coverslips at a density of 2x10^3^ cells/coverslip and cultured for 24 h at 37°C. MSCs were pretreated with 50 μM EdU working solution for 24 h and fixed in 4% PFA for 20 min at room temperature. Click-iT reaction mixture was added and incubated away from light for 30 min, washed with PBS, and Hoechst 33342 (Thermo Fisher Scientific, USA) was used to stain the nuclei for 15 min. Fluorescence imaging was observed by a fluorescence confocal microscopy (Nikon, Japan).

ALP staining assay: ALP staining assay was performed using the Alkaline Phosphatase Assay Kit (Beyotime, China). Briefly, the 3rd-passaged MSCs were seeded on the 6-well plate at a density of 1x10^6^ cells/well and cultured for 48 h at 37°C. After washing with PBS, MSC was fixed with PFA for 15 min, and then staining working solution was added and incubated for 5 min at 37°C. The reaction was terminated by adding ddH_2_O and photographed under the microscope.

Alizarin red S staining assay: For Alizarin red S staining assay, the 3rd-passaged MSCs were seeded on a 12-well plate at a density of 5x10^5^ cells/well and cultured for 48 h at 37°C. When cells reached 90% confluency, the medium was changed to the osteogenic induction culture α-MEM medium (Gibco, USA) supplemented with 5% FBS (ExCell Bio, China), 10 mM β-glycerophosphate, 50 μg/ml Vitamin C and 10 nM dexamethasone (all from Sigma-Aldrich, USA). After 28 days, cells were fixed with 4% PFA for 20 min, stained with Alizarin red S (Sigma-Aldrich, USA), and photographed under the light microscope.

Oil red O staining assay: For Oil red O staining assay, the 3rd-passaged MSCs were seeded on a 12-well plate at a density of 5x10^5^ cells/well and cultured for 48 h at 37°C. When cells reached 90% confluency, the medium was changed to the adipogenic induction culture α-MEM medium (Gibco, USA) supplemented with 0.2 mM indomethacin, 0.5 mM isobutylmethylxanthine, 10 μM insulin and 1 μM dexamethasone (all from Sigma-Aldrich, USA). After 28 days, cells were fixed with 4% PFA for 20 min, stained with oil red O (Sigma-Aldrich, USA), and photographed under the light microscope.

SA-β-gal staining assay: SA-β-gal staining assay was performed using the Senescence β-Galactosidase Staining Kit (Beyotime, China). Briefly, the 3rd-passaged MSCs were seeded on a 12-well plate at a density of 5x10^5^ cells/well and cultured for 48 h at 37°C. After washing with PBS, MSCs were fixed with 4% PFA for 20 min, and then staining working solution was added and incubated overnight at 37°C without CO_2._

Flow cytometric assay: Flow cytometric assay was performed as previously described. Briefly, the 3rd-passaged MSCs were collected, and no less than 2x10^5^ cells were counted and resuspended with PBS. MSCs were incubated with FITC-conjugated anti-CD73 (127220, Biolegend, USA; diluted 1:100), PE-conjugated anti-CD90 (12-0909-41, eBioscience, USA; diluted 1:100), PE-conjugated anti-CD105 (PA5-114983, Biolegend, USA; diluted 1:100), PE-conjugated anti-CD34 (343506, Biolegend, USA; diluted 1:100), or PE-conjugated anti-CD45 (304008, Biolegend, USA; diluted 1:100) at 4°C for 30 min. The percentages of positive cells were analyzed by a CytoFLEX flow cytometer (Beckman Coulter, USA).

**Extraction and isolation of Golgi fraction**

The Golgi fraction was extracted from tissues using a Golgi extraction kit (BaiAoLaiBo, China). Briefly, 0.1 g tissues were minced at 4°C, followed by the addition of 500 μl of reagent A to resuspend the tissue, which was then placed on ice for 10 min. The tissues were homogenized several times with a homogenizer, and the supernatant was centrifuged at 1000 g for 5 min at 4°C, then at 3000 g for 10 min at 4°C, and finally at 5000 g for 10 min at 4°C. Subsequently, 10 μl of WT reagent was added to the precipitate, which was then centrifuged at 4°C and 20,000 g for 20 min. After adding 500 μl of reagent B to resuspend the precipitate, the supernatant was discarded by centrifugation at 4°C and 20,000 g for 30 min. The Golgi fraction was then obtained and dissolved in culture medium to treat BMSCs at 20 μg/ml of protein concentration.

**Transfection of siRNAs**

MSCs was transfected with siRNA-negative control (si-NC) or siRNA-STX5 (GenePharma, China) using a transfection kit (GenePharma, China) at a final concentration of 75 pM. Transfection efficiency was measured 24 h after transfection *via* qRT-PCR. The MSCs transfected with siRNAs for 72 h were used for subsequent experiments.

**Collection and treatment of BMS**

Mouse femora and tibiae were isolated on an ultra-clean bench, and the bone marrow cavity was repeatedly rinsed with 1 ml PBS per mouse. The suspension was centrifuged at 500 g for 10 min, continued to be centrifuged at 2000 g for 10 min, and further centrifuged at 16000 g for 30 min to obtain BMS. BMS was mixed with culture medium at 1:5 and added to MSCs during the experimental period.

**BMT procedures**

Mice were anesthetized by intraperitoneal injection of 1% pentobarbital sodium. The femur was exposed after bluntly separating the fascia and muscle tissue along the long axis, and a defect with an area of 4 mm^2^ (4 mm long x 1 mm wide) was prepared in the mid-femur using a dental turbine. After saline rinsing, CA (0.3 mg in protein amount per mouse) or CA-EVs (150 μg in protein amount per mouse) mixed with the Pluronic F-127 gel (Sigma-Aldrich, USA) were placed in the defect location and protected by applying absorbable biofilm cover. In the control group, only absorbable biofilm and gel were placed. The muscle and skin were sutured layer by layer, and 1% iodophor was applied to prevent infection.

**Fluorescent tracing of CA-EVs**

For CA-EV uptake by BMSCs *in vitro*, the 2nd-passaged BMSCs were seeded on coverslips at a density of 1x10^5^ cells and cultured for 48 h at 37°C. CA-EVs were labeled with PKH26 (Sigma-Aldrich, USA) following the instruction, resuspended by culture medium, and added to cultured BMSCs for 8 h. After washing with PBS, BMSCs were fixed with 4% PFA for 20 min, blocked at 37°C for 30 min, and incubated with a fluorescent phalloidin probe for F-actin (Invitrogen, USA; diluted 1:100) at room temperature for 45 min. BMSCs were washed with PBS, and stained with Hoechst 33342 (Sigma-Aldrich, USA) for 15 min. Fluorescence imaging was observed by a confocal microscopy.

For CA-EV uptake by BMSCs *in vivo*, CA-EVs were labeled with PKH26 and premixed with the Pluronic F-127 gel (Sigma-Aldrich, USA) at 4°C. Then, the bone defect model was prepared as stated below, and CA-EVs were placed in the defect for 72 h. After 4% PFA fixation and 17% ethylene diamine tetraacetic acid (EDTA) (Sigma-Aldrich, USA) decalcification, the femoral tissue was prepared into 12 μm sections and stained for MSC markers. Fluorescence imaging was observed by a confocal microscopy.

For biodistribution analysis, CA-EVs were labeled with the fluorescent lipophilic tracer DiR (Yeasen, China) or PKH26, and were then injected intravenously into mice for 24 h. The biodistribution imaging *in vivo* was obtained by IVIS Spectrum (PerkinElmer, USA). For tracing of CA-EVs in target organs, samples were fixed, cryoprotected by sucrose and frozen-sectioned. Slices were stained with Hoechst 33342 (Sigma-Aldrich, USA) and observed by a confocal microscopy.

**Construction of GFP-MSC-CA and analysis of GFP-EVs after implantation**

The 3rd-passaged SHED were seeded on 6-well plates at a density of 1x10^6^/well and cultured for 24 h at 37°C. After attachment, the cells were replaced with antibiotic-free and serum-free culture medium, and then lentiviral transfection reagents (GeneChem, China) were added and were incubated for 48 h followed by observation by a fluorescence microscopy. GFP-CA was constructed accordingly and implanted in the femoral bone defects of mice. After 6 days, the femora were isolated, and the whole bone marrow was flushed out using PBS. Whole bone marrow EVs were extracted under dark conditions, and the proportion of GFP positive population was analyzed using flow cytometry.

**Micro-CT analysis**

After sacrifice, femora were collected and fixed in 4% PFA. The femur samples were scanned by micro-CT (ALWAYS IMAGING, China) at a resolution of 7.29 μm, voltage of 70 kV, and a current of 60 μA. The Mimics 13.0 software was used to analyze the data with quantification of trabecular bone parameters after three-dimensional reconstruction.

**Histological analysis**

Femur bone tissue or CA samples was fixed in 4% PFA for 8 h and immersed in 17% EDTA (Sigma-Aldrich, USA) at 4°C for 7 days. Gradient alcohol treatment of the tissues was performed followed by being embedded in paraffin and prepared into 4 μm femur bone sections. Sections were stained for H&E using a commercial kit (Leica, Germany). Masson’s trichrome staining was performed using the Masson’s trichrome staining kit (Baso, China). TRAP staining was performed using the 387A-1KT commercial kit (Sigma-Aldrich, USA) counteracted with 1% Methyl Green Stain Solution (Solarbio, China). After staining, sections were sealed with neutral balsam (Sigma-Aldrich, USA) and photographed under the light microscope.

**IF staining**

Femur samples were fixed with 4% PFA for 8 h, washed in PBS, and then decalcified in 17% EDTA for 7 days. Tissues were dehydrated in freshly prepared sucrose solution of 30% mass concentration for 8 h and embedded in the OCT compound (Leica, Germany) to prepare 12 μm femur tissue sections. The sections were then permeabilized with 0.3% TritonX-100 (Sigma-Aldrich, USA), blocked with serum for 30 min at 37°C, and primary antibodies were added and incubated for 8 h at 4°C. The sections were washed with PBS and incubated with the secondary antibodies for 2 h at 37°C. After washing with PBS, the sections were counterstained by Hoechst 33342 for 15 min at room temperature. The IF staining antibodies used are as follows: Ki67 (AB15580, Abcam, USA; diluted 1:200), GM130 (610822, BD, USA; diluted 1:200), RUNX2 (12556, Cell Signaling Technology, USA; diluted 1:200), CD44 (PA5-114983, Invitrogen, USA; diluted 1:200), CD73 (127220, Biolegend, USA; diluted 1:200), and CD105 (MAB1320, R&D system, USA; diluted 1:200).

**RNA isolation and qRT-PCR analysis**

Extraction of total RNA using the Trizol reagent (Thermo Fisher Scientific, USA) method was performed as reported. Reverse transcription of RNA to cDNA was performed using 5x PrimeScript RT Master Mix (Takara, Japan). qRT-PCR was performed using the SYBR Premix Ex TaqTMⅡ (Takara, Japan), and the signal was detected with a Real-Time (Bio-Rad, USA) system. The primer sequences used in this study are listed in Table S6.

**Western blot analysis**

Western blot analysis was performed as previously reported. Briefly, protein samples were prepared using lysis buffer (Beyotime, China), and protein concentration was quantified using the BCA Protein Assay kit (Beyotime, China). Protein samples at a concentration of 1 mg/ml were loaded into 4%-20% SDS-PAGE gels (EpiZyme, China) to separate proteins and then transferred onto polyvinylidene fluoride (PVDF) membranes (Roche, Switzerland). The PVDF membrane was blocked with 5% skim milk (AR, China) at room temperature for 2 h and then incubated with primary antibodies at 4°C for 16 h. After washing with TBST (AR, China), the PVDF membranes were incubated with secondary antibodies at room temperature for 2 h. Expression of the protein was detected by the gel imaging system (Tanon, China) after the PVDF membranes were soaked into an enhanced chemiluminescence solution (AR, China). The antibodies used in this study are as follows: RUNX2 (12556, Cell Signaling Technology, USA; diluted 1:1000), SP7 (SC-22538, Santa Cruz Biotechnology, USA; diluted 1:500), ALP (SC-79840, Santa Cruz Biotechnology, USA; diluted 1:500), STX5 (14151S, Cell Signaling Technology, USA; diluted 1:1000), STX16 (AB134945, Abcam, USA; diluted 1:1000), USO1 (A20950, ABclonal, China; diluted 1:200), GALT (A21426, ABclonal, China; diluted 1:1000), GM130 (610822, BD, USA; diluted 1:1000), TGN38 (SC-166594, Santa Cruz Biotechnology, USA; diluted 1:500), α-SNAP (A19227, ABclonal, China; diluted 1:500), NSF (A0926, ABclonal, China, diluted 1:1000), GRASP55 (SC-271840, Santa Cruz Biotechnology; USA; diluted 1:500), GRASP65 (SC-374423, Santa Cruz Biotechnology, USA; diluted 1:500), VAMP3 (NB300-510, Novus, USA; diluted 1:1000), VAMP4 (PA1-768, Invitrogen, USA; diluted 1:1000), β-actin (CW0096A, Cwbiotech, China; diluted 1:1000), GAPDH (30201ES20, Yeasen, China; diluted 1:1000), CD9 (AB236630, Abcam, USA; diluted 1:1000), CD63 (AB271286, Abcam, USA; diluted 1:1000), TSG101 (AB125011, Abcam, USA; diluted 1:1000), BMP2 (AB114933, Abcam, USA; diluted 1:1000), BMP4 (AB39973, Abcam, USA; diluted 1:1000), p-β-catenin (8480s, Cell Signaling Technology, USA; diluted 1:1000), β-catenin (A19657, ABclonal, China; diluted 1:1000), Axin2 (A2513, ABclonal, China; diluted 1:1000), p-GSK-3β (SC-11757, Santa Cruz Biotechnology, USA; diluted 1:1000), GSK-3β (A16868, ABclonal, China; diluted 1:1000), Cyclin D1 (AB134175, Abcam, USA; diluted 1:1000), Cyclin E2 (SC-28351, Santa Cruz Biotechnology, USA; diluted 1:1000), Golgin84 (NBP1-83352, Nova Biologicals, USA; diluted 1:1000) and secondary antibodies (Jackson ImmunoResearch, USA).

**RNA-seq analysis**

Total RNA extraction was performed using Trizol (Thermo Fisher Scientific, USA). A total amount of 1 μg RNA per sample was used as the input material for RNA sample preparations. Sequencing libraries were generated using NR604-VAHTS® Fast RNA-seq Library Prep Kit for Illumina (Vazyme, China) following manufacturer’s recommendations, and index codes were added to attribute sequences to each sample. PCR products were purified, and library quality was assessed on the Agilent Bioanalyzer 2100 system. The library preparations were sequenced on an Illumina Novaseq 6000 platform and paired-end reads were generated. For samples with biological replicates, differential expression analysis was performed using the DESeq R package. DESeq provides statistical routines for determining differential expression in digital gene expression data using a model based on the negative binomial distribution. The resulting *P*-values were adjusted using Benjamini and Hochberg’s approach for controlling the false discovery rate. The *P*-value was adjusted using the *Q*-value. The *Q*-value < 0.05 & | log_2_ (foldchange）|> 1 was set as the threshold for significantly differential expression. For GO enrichment analysis, the DEGs were mapped to each term of the GO database, the number of DEGs per term was counted, and then a hypergeometric test was applied. KEGG and PPI analyses were accordingly performed.

**Proteomic analysis**

For protein extraction, 300 μl of lysis buffer was added to the samples. The supernatant was collected after centrifugation at 14,000 g for 20 min, and protein concentration was determined using the BCA method. A 50 μg aliquot of protein extracted from each sample was then subjected to reduction. 10 μg of each sample was subjected to SDS-PAGE electrophoresis, stained with Caumas Brilliant Blue for 30 min, and decolorized until the background was clear. Nanoflow LC-MS/MS analysis of tryptic peptides was conducted on a quadrupole Orbitrap mass spectrometer (Thermo Fisher Scientific, USA) coupled to an EASY nLC 1200 ultra-high-pressure system (Thermo Fisher Scientific, USA) *via* a nano-electrospray ion source. All RAW files were analyzed using the Proteome Discoverer suite (Thermo Fisher Scientific, USA). GO annotation of the proteome was derived from the GO database. Proteins were classified by GO annotation based on three categories as follows: Molecular Function, Biological Process, and Cellular Component. The protein domain functional descriptions identified in this study were annotated by Pfam. The pathway analysis was performed using the KEGG pathway.

**Supplementary Figures and Figure Legends**


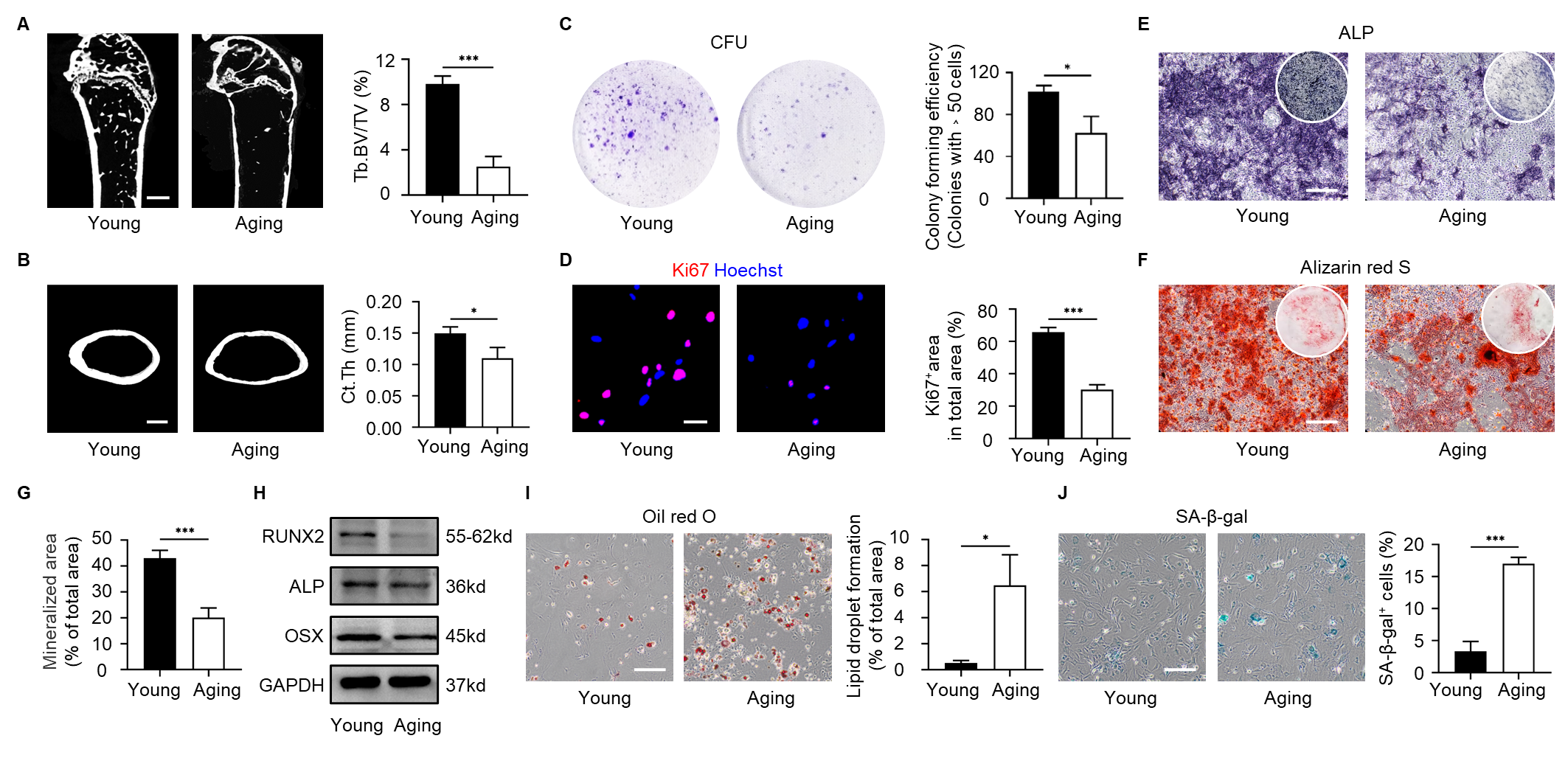


**Figure S1. Bone aging phenotype and functional characterization of aging BMSCs.**

(A and B) Micro-CT analysis of femoral bone mass and cortical bone thickness. Bars: 250 μm (A) and 50 μm (B). N = 3 per group. (C and D) Colony formation and Ki67 IF staining with the quantification. Bar: 50 μm. N=3 per group. (E and F) ALP and Alizarin red S staining of osteogenesis-induced BMSCs. Bars: 200 μm. (G) The quantification of mineralized area over total area in Alizarin red S staining. N = 3 per group. (H) Western blot analysis of osteogenesis-related protein expression in cultured BMSCs. (I) Oil red O staining of adipogenesis-induced BMSCs and the quantification. Bar: 200 μm. N = 3 per group. (J) SA-β-gal staining demonstrating cellular senescence of BMSCs and the quantification. Bar: 200 μm. N = 3 per group. Data are presented as Mean ± SD. Statistical analyses were performed by Student’s *t* test. **P* < 0.05; ****P* < 0.001.


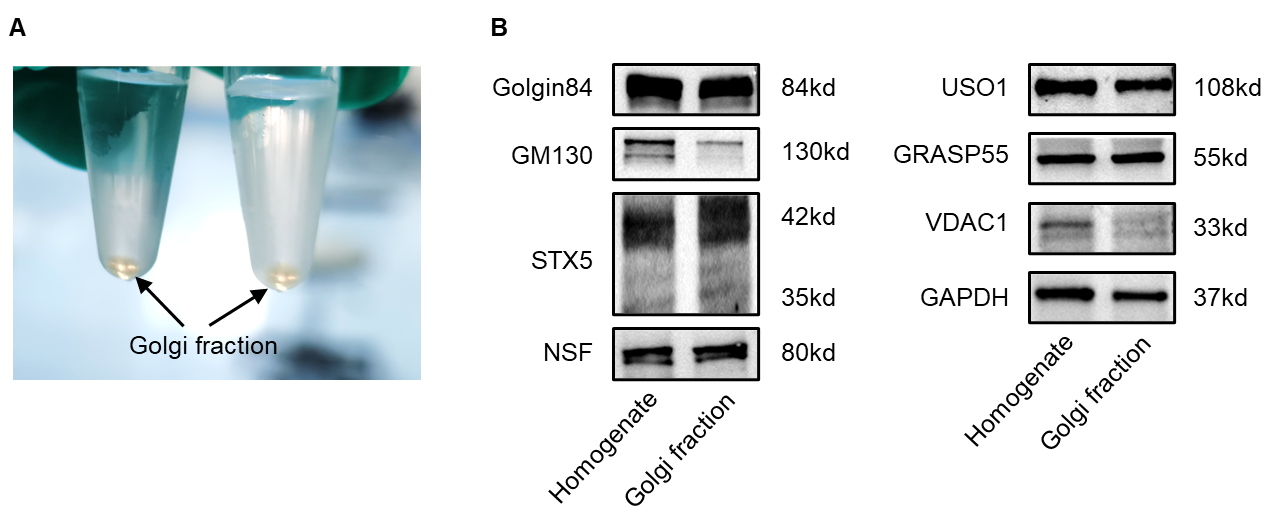


**Figure S2. Extraction and characterization of Golgi fraction.**

(A) Morphology of Golgi fraction observed in centrifuge tubes. (B) Western blot analysis of Golgi structure protein and Golgi vesicle trafficking-related protein expression in Golgi fraction and tissue homogenates.


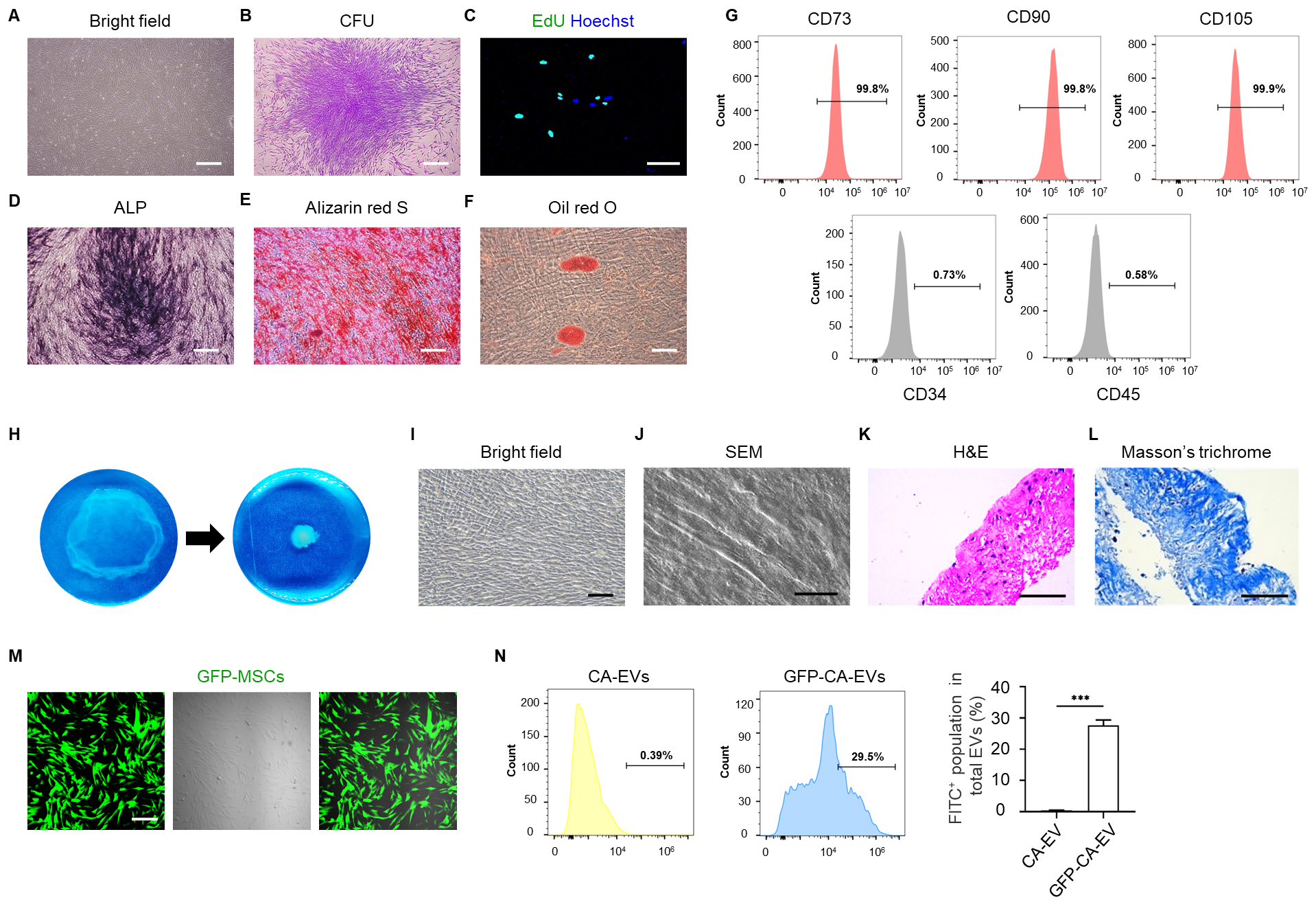


**Figure S3. Construction of CA and characterization of GFP-CA-EVs.**

(A) Morphology of MSCs observed under light microscope. Bar: 500 μm. (B and C) Colony formation and EdU staining of MSCs. Bar: 200 μm (B) and 100 μm (C). (D and E) ALP and Alizarin red S staining of osteogenesis-induced MSCs. Bars: 200 μm. (F) Oil red O staining of adipogenesis-induced MSCs. Bar: 200 μm. (G) Flow cytometric analysis of surface markers of MSCs. (H) Morphology of CA observed in well plates. (I) Morphology of CA observed under light microscope. Scale bar: 200 μm. (J) Representative SEM image showing the morphology of CA. Bar: 25 μm. (K) H&E staining of the structure of CA. Bar: 75 μm. (L) Masson’s trichrome staining of the structure of CA. Bar: 75 μm. (M) Fluorescence and bright field images of GFP-MSCs. Scale bar: 200 μm. (N) Flow cytometric analysis of GFP^+^ population expression in total CA-EVs. Data are presented as Mean ± SD. Statistical analyses were performed by Student’s *t* test. ****P* < 0.001.


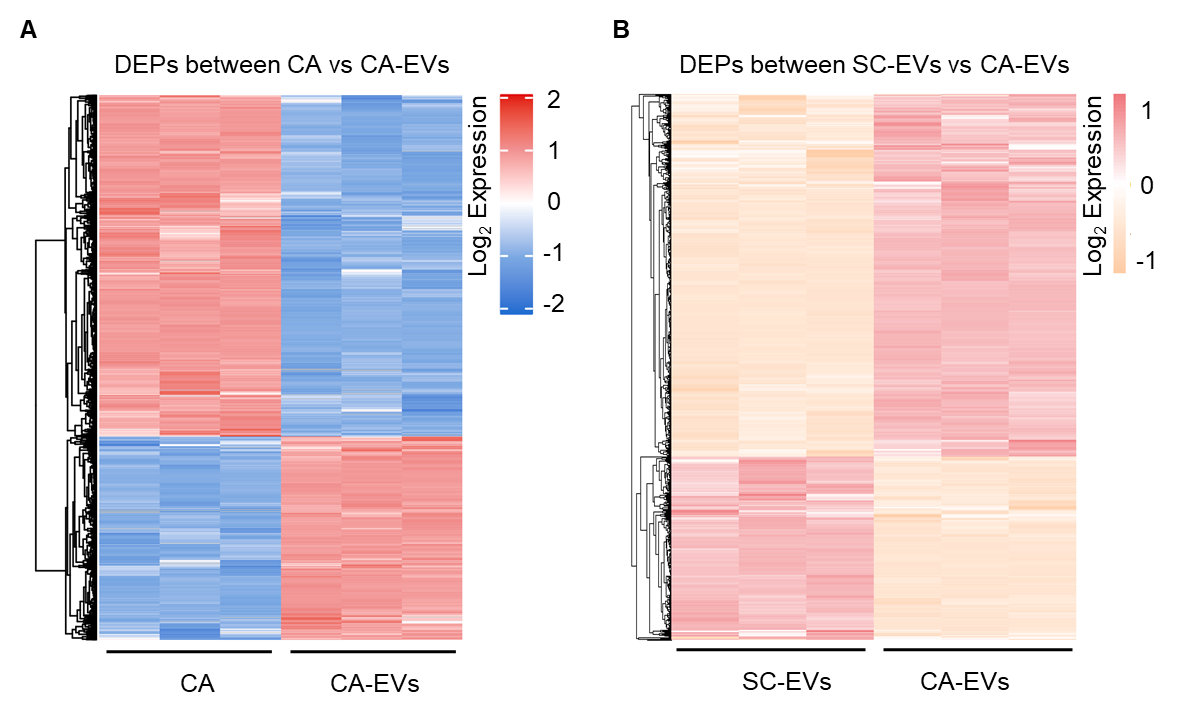


**Figure S4. Proteomic analysis of CA vs CA-EVs and SC-EVs vs CA-EVs.**

(A) Hierarchical clustering of DEPs between CA and CA-EVs. (B) Hierarchical clustering of DEPs between SC-EVs and CA-EVs.


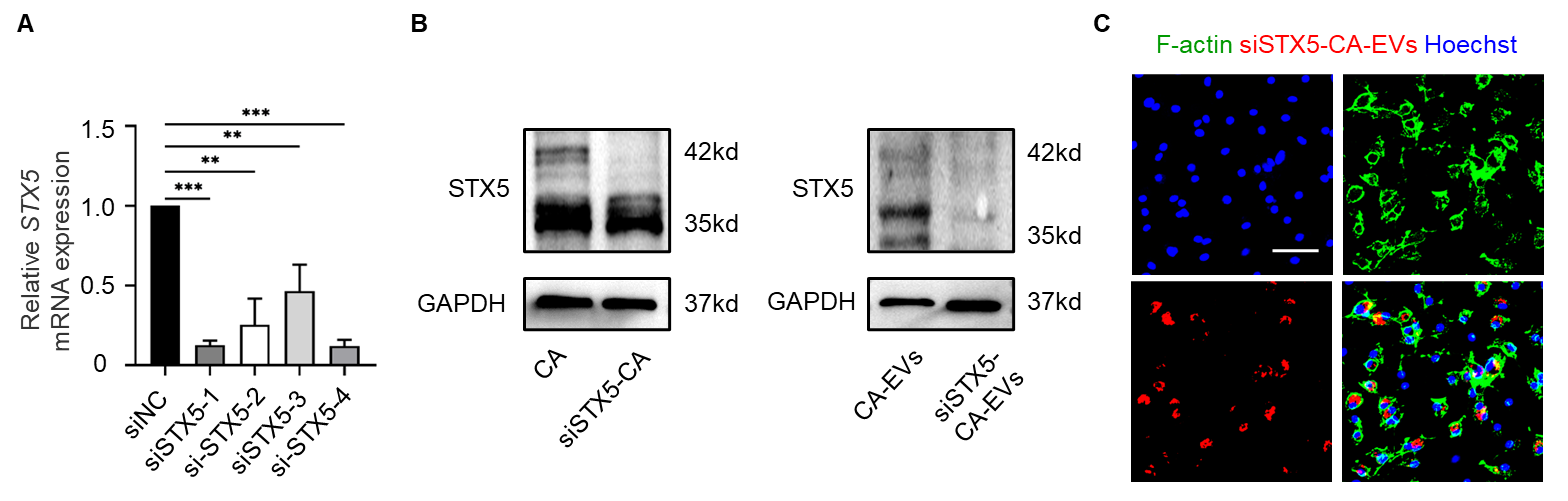


**Figure S5. Identification of knockdown efficiency of STX5.**

(A) qRT-PCR analysis of mRNA expression levels of STX5. N = 4 per group. (B) Western blot analysis of STX5 protein expression in CA and CA-EVs. (C) Images showing siSTX5-CA-EVs phagocytosed by BMSCs *in vitro*. Bar: 50 μm. Data are presented as Mean ± SD. Statistical analyses were performed by one-way ANOVA. ***P* < 0.01; ****P* < 0.001.


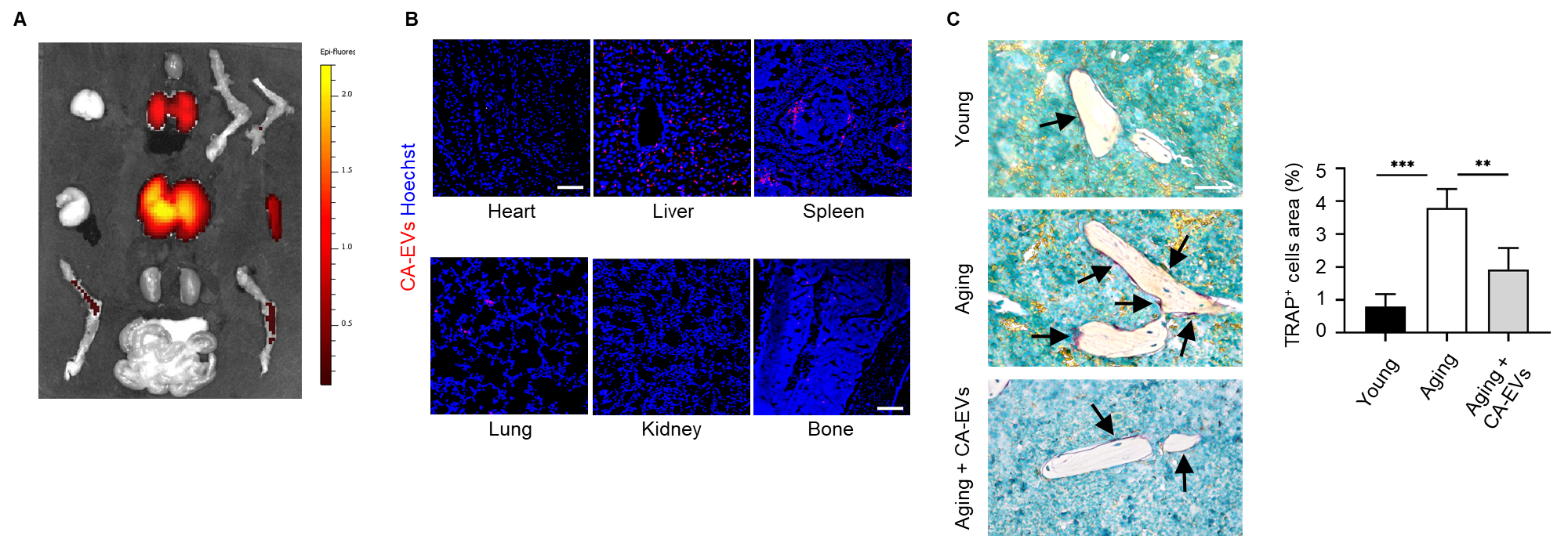


**Figure S6. CA-EV infusion attenuates bone resorption in aging mice.**

(A and B) Live animal imaging and fluorescence tracking of the *in vivo* distribution of infused CA-EVs. Bars: 100 μm / 200 μm (bone). (C) After 6 weeks of treatment with CA-EVs, TRAP staining showing the femoral osteoclasts and the quantification. Data are presented as Mean ± SD. Statistical analyses were performed by one-way ANOVA. ***P* < 0.01; ****P* < 0.001.


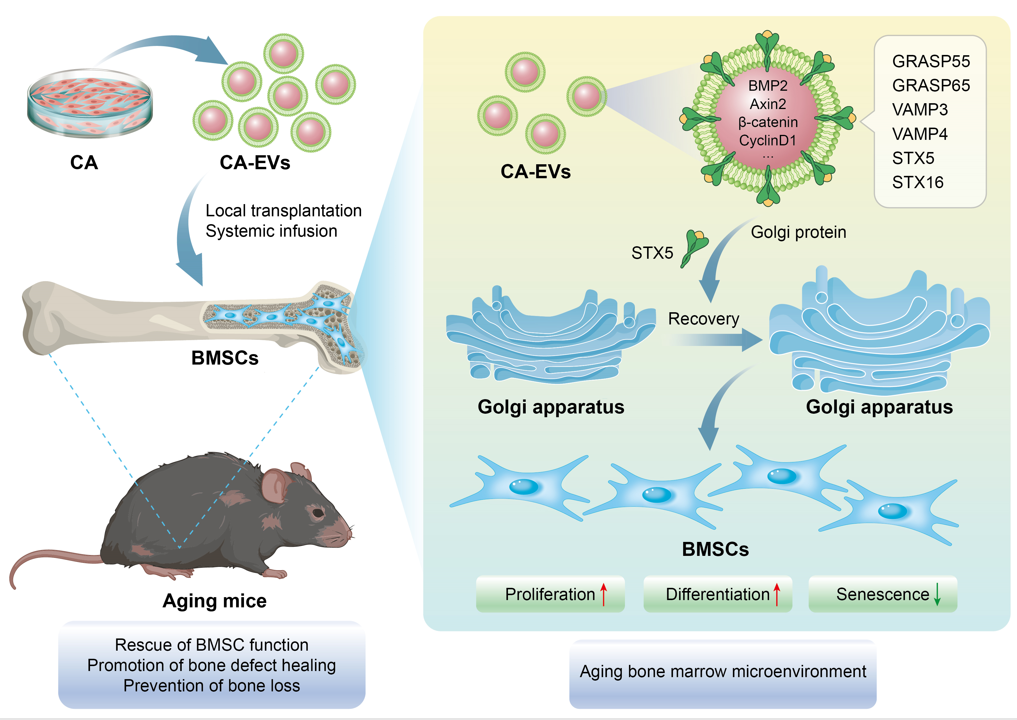


**Figure S7. Graphical conclusion of this study.**

Aging bone microenvironment impairs the Golgi apparatus and diminishes bone marrow mesenchymal stem cell (BMSC) function and regeneration. Interestingly, replenishment of cell aggregates-derived extracellular vesicles (CA-EVs) rescues Golgi dysfunction and empowers senescent BMSCs. Importantly, *in vivo* administration of CA-EVs significantly enhanced the rate of bone defect repair and improved bone mass in aging mice, suggesting their therapeutic value for treating age-related osteoporosis and promoting bone regeneration.

**Supplementary Table Captions**

**Table S1.** List of all the proteins identified during proteomic analysis in Figure 1A and B.

**Table S2.** List of all the proteins identified during transcriptomic analysis in Figure 1L-N.

**Table S3.** List of all the proteins identified during transcriptomic analysis in Figure 3H-L.

**Table S4.** List of all the proteins identified during proteomic analysis in Figure 4E and F.

**Table S5.** List of all the proteins identified during proteomic analysis in Figure 4G and H.

**Table S6.** The primer sequences used in this study.
